# Cardiovascular effects of a selective 5-HT4 agonist and an alpha-2 adrenoceptor antagonist in etorphine immobilised sheep (*Ovis aries*) - a randomised, prospective, and controlled trial

**DOI:** 10.1186/s12917-026-05379-x

**Published:** 2026-03-06

**Authors:** Hathaipat Rattanathanya, Anna Binetti, Friederike Pohlin, Susana C.M. Ferreira, Christina Braun, Johannes Schramel, Leith C.R. Meyer, Anna Haw, Marja Raekallio, Stefan Böhmdorfer, Szilvia Kalogeropoulu, Martina Mosing, Gabrielle Stalder

**Affiliations:** 1 Research Institute of Wildlife Ecology, Department of Interdisciplinary Life Sciences, University of Veterinary Medicine Vienna, Vienna, Austria; 2 Chulabhorn Royal Academy, Bangkok, Thailand; 3 Anaesthesiology and Intensive-Care Medicine, Clinical Centre for Small Animal Health and Research, Clinical Department for Small Animals and Horses, University of Veterinary Medicine Vienna, Vienna, Austria; 4 Department of Production Animal Studies and Centre for Veterinary Wildlife Research, Faculty of Veterinary Science, University of Pretoria, Pretoria, South Africa; 5 Animal Welfare Science and Bioethics Centre, School of Veterinary Science, Massey University, Palmerston North, New Zealand; 6 Brain Function Research Group, Department of Physiology, School of Biomedical Sciences, University of the Witwatersrand, Johannesburg, South Africa; 7 Faculty of Veterinary Medicine, Department of Equine and Small Animal Medicine, University of Helsinki, Helsinki, Finland; 8 Institute of Chemistry of Renewable Resources, BOKU University, Vienna, Austria

**Keywords:** BIMU-8, Etorphine, Opioid, Vatinoxan, Pulmonary hypertension, Cardiovascular

## Abstract

**Background:**

Etorphine, a highly potent opioid widely used in wildlife immobilisation, is known to cause cardiorespiratory compromise. This study aimed to investigate the effects of a serotonergic agonist, BIMU-8 and an alpha-2 adrenoreceptor antagonist, vatinoxan on pulmonary hypertension and cardiovascular function in etorphine-immobilised sheep, as a model for wild ungulates. Six sheep were immobilised three times in a randomised, prospective, controlled crossover design using intramuscular etorphine (0.05 mg·kg^−1^). Seven minutes later, sheep received intravenous BIMU-8 (1.5 mg·kg^−1^), vatinoxan (0.15 mg·kg^−1^), or sterile water (control). Respiratory rate, pulmonary arterial, systemic arterial and central venous pressures, electrocardiography, heart rate, and cardiac output were recorded. Data were collected at resting state, six minutes post-etorphine, and at six-minute intervals post-treatment. Naltrexone was administered 19 minutes post-treatment to reverse immobilisation. Linear mixed-effects models were used for statistical analysis.

**Results:**

Etorphine induced bradypnea, pulmonary hypertension, dysrhythmias, and tachycardia in sheep. BIMU-8 significantly reduced mean pulmonary arterial pressure (F_2,91_ = 4.17, *p* = 0.02) and mean arterial pressure (F_2,89_ = 6.01, *p* < 0.01) but was associated with severe tachydysrhythmia. Vatinoxan decreased respiratory rate (F_2,91_ = 4.13, *p* < 0.01), increased cardiac output (F_2,91_ = 10.88, *p* < 0.01), and reduced body temperature (F_6,85_ = 2.2, *p* = 0.05), while having no effect on pulmonary or systemic arterial pressures.

**Conclusion:**

BIMU-8 reduces etorphine-induced pulmonary and systemic hypertension, but causes tachydysrhythmias, requiring further evaluation. Vatinoxan improves cardiac output, without alleviating pulmonary hypertension in etorphine-immobilised sheep.

**Supplementary Information:**

The online version contains supplementary material available at 10.1186/s12917-026-05379-x.

## Background

In wildlife anaesthesia, potent opioids such as etorphine, are essential for immobilising large wild herbivores [1, 2]. Etorphine provides rapid, reliable, and reversible immobilisation while requiring only a small application volume [3]. However, despite its desirable immobilising effects, etorphine also causes significant physiological dysfunction, with respiratory and cardiovascular impairment causing hypoxia being arguably the most severe and life-threatening side effect [4, 5].

The hypoxia is caused by a decreased central respiratory drive due to the direct effect of mu-opioid receptor activation in the brainstem, together with a suppression of the peripheral hypoxic and hypercapnic respiratory drive via chemoreceptors [6–8]. Moreover, indirect opioid-induced haemodynamic changes such as vasoconstriction in the pulmonary vasculature, causing pulmonary hypertension, also play an important role in the pathophysiology of hypoxia in etorphine-immobilised animals [4, 9] .

Several approaches have been investigated to alleviate opioid-induced hypoxia, including the administration of serotonin receptor agonists, which have shown promising results in mitigating respiratory depression [10–12]. BIMU-8, a selective 5-hydroxytryptamine 4 (5-HT4) receptor agonist, increased respiratory rate (fR), reduced the alveolar-arterial pressure gradient (A–a gradient) and systemic blood pressure, while elevating heart rate (HR) in etorphine-immobilised goats [13]. These effects suggest that BIMU-8 may alleviate etorphine-induced pulmonary hypertension and hypoxia while maintaining its analgesic and sedative effects.

Etorphine has been described to induce systemic hypotension or hypertension, bradycardia or tachycardia, and alterations in cardiac output (CO) [4, 9, 14–16]. These effects are potentially caused by endogenous catecholamine release, either directly via mu-opioid receptor activation or indirectly through various stimuli such as hypoxia, hypercapnia, or the mounting of a stress response [4, 9, 14].

Vatinoxan, a peripherally acting alpha-2 adrenoceptor antagonist, has gained interest because of its ability to mitigate the adverse cardiopulmonary effects of different alpha-2 adrenoceptor agonists, such as systemic hypertension and bradycardia, while preserving centrally mediated sedation [17]. In various species, including sheep anaesthetised with alpha-2 agonists, vatinoxan improved cardiovascular function by reducing systemic blood pressure and peripheral vascular resistance [18–23]. By blocking postsynaptic pulmonary alpha-2 adrenoceptors, vatinoxan may counteract etorphine-induced pulmonary vasoconstriction and hypertension, thereby reducing the risk of hypoxia.

The aim of this study was to evaluate the cardiovascular effects of the novel drugs BIMU-8 and vatinoxan in etorphine-immobilised sheep, serving as a model for wild ungulates. We hypothesised that BIMU-8 would enhance respiratory drive and induce vasodilation in both the pulmonary and systemic circulation, while vatinoxan would alleviate pulmonary hypertension and improve tissue perfusion by counteracting etorphine-induced pulmonary and systemic vasoconstriction.

## Methods

### Animals

Based on prior studies and an *a priori* power analysis, six animals per group were determined to be sufficient to detect a physiologically meaningful 7–10 mmHg difference in mean pulmonary arterial pressure (mPAP), assuming a standard deviation of 3–5 mmHg, a significance level of 0.05, and at least 95% statistical power. Two additional animals per group were included as reserves, resulting in a total of eight animals per group. Therefore, eight adult female sheep (*Ovis aries*), with a median age of 5.9 ± 0.3 years and a mean body weight of 92.9 ± 9.5 kg, were kept in a 1 ha outdoor enclosure with shelter at the Research Institute of Wildlife Ecology of the University of Veterinary Medicine, Vienna, Austria. All animals were institution-owned and sourced from the VetFarm of the University of Veterinary Medicine, Vienna, Austria. Their diet consisted of hay, supplemented with sheep concentrate pellets (Alpenkorn, Garant-Tiernahrung Gesellschaft m.b.H., Pöchlarn, Austria), and water was provided *ad libitum*. Over a three-month period, the sheep underwent habituation and training to facilitate handling and standing in a custom-made sling (TBTN^®^. GTRD^®^, Zürich, Switzerland).

The sheep were deemed healthy based on complete blood count, and serum biochemistry analyses performed 3 weeks before the start of the experiments. The sheep were transported to the research facility one week prior to the start of the experiments for acclimatisation in order to minimise handling-induced sympathetic stress responses during data collection. At the research facility, the sheep were housed in two 4 × 4 m stalls, each accommodating four animals. Bedding consisted of wood shavings and straw and was replaced daily. Animals had unrestricted access to hay and water at all times. On the day before each experiment, a physical examination was performed to confirm good health. Six healthy, well-trained sheep were included in the study, while the two additional animals were kept as reserves.

Following the completion of all experimental procedures, animals were assessed by veterinary staff and transferred into private care through the institution’s approved rehoming program. No animals were euthanised for the purposes of this study.

### Instrumentation

**Fig. 1:**
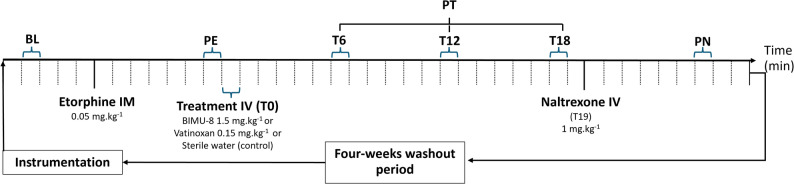
Experimental timeline. Each sheep (*n *= 6) received three treatments; BIMU-8, vatinoxan or sterile water (control) in a randomised, controlled, crossover trial with a four-week washout period between treatments. Animals were instrumented before baseline measurements (BL) assessing the following variables at each timepoint: respiratory rate (fR), pulmonary arterial pressure (PAP), arterial pressure (AP), central venous pressure (CVP), ECG, heart rate (HR), cardiac output (CO), and body temperature (BT). Etorphine IM: administration of 0.05 mg·kg^−1^ etorphine hydrochloride IM, Post etorphine (PE) = measurements after etorphine administration; (T0) Treatment IV = administration of 1.5 mg·kg^−1^ BIMU-8, 0.15 mg·kg^−1^ vatinoxan or sterile water equal in volume to the amount of other treatments in mL (control) IV over one minute; timepoint; (PT at T6, T12, T18) = measurements after treatments; Naltrexone IV = administration of 1 mg·kg^−1^ naltrexone (T19). Post naltrexone (PN) = measurements after 6 minutes of naltrexone administration

The experiments were conducted in a large-animal surgery room at the Equine Clinic of the University of Veterinary Medicine, which was maintained at a constant temperature (22.2 ± 2.8 °C). Sheep were fasted for 16 hours before the start of the experiment to reduce the risk of bloating. Before being brought into the surgery room, the sheep were weighed, and a local anaesthetic cream, containing 25 mg lidocaine and 25 mg prilocaine per gram (Emla 5%, AstraZeneca Pharmaceuticals (Pty) Ltd., Wilmington, DE, USA), was applied to both the clipped ears and clipped neck area. Upon arrival in the experimental room, each animal was guided onto the customised sling connected to a ceiling-mounted crane in the surgery room. The sling supported the sheep’s body weight and maintained it in a standing position, with the head held upright throughout immobilisation (see Fig 1. in supplementary materials, S1). Thereafter, midazolam (0.3 mg·kg^−1^; Midazolam 5%, Oberösterreichische Gesundheitsholding GmbH, Linz, Austria) was injected intravenously (IV) to allow for instrumentation.

After aseptic preparation, of the skin 2 mL of lidocaine (Xylanaest^®^ purum 2%, Gebro Pharma GmbH, Fieberbrunn, Austria) were injected subcutaneously at the mid-neck region to desensitise the skin and underlying tissues. An 8.5 Fr percutaneous sheath introducer set with 10 cm sheath (Teleflex^®^, Arrow international LLC, Morrisville, NC, USA) was then inserted into the left jugular vein, through which a continuous cardiac output thermodilution catheter (Swan-Ganz™ Catheter, Edwards Life Sciences, Irvine, CA, USA) was advanced in the vein, the right ventricle and pulmonary artery under pressure guidance. The Swan-Ganz catheter was connected to a pre-calibrated pressure transducer (Disposable transducer, B.Braun Melsungen AG, Melsungen, Germany) and amplifier via three-way stopcocks to measure pulmonary arterial pressure (PAP) and central venous pressure (CVP).

After intradermal infiltration with 0.1 mL of lidocaine, an 18-gauge intravenous catheter (Vasofix^®^ Safety, B.Braun Melsungen AG, Melsungen, Germany) was placed into one of the auricular arteries and connected via a fluid-filled non-compliant tubing system to a pre-calibrated pressure transducer (Disposable transducer, B.Braun Melsungen AG, Melsungen, Germany).

Both transducers, connected to the Swan-Ganz catheter and the auricular arterial catheter, were secured at the level of the heart base (scapulohumeral joint) and zeroed to atmospheric pressure.

PAP, CVP and arterial blood pressure (AP) were measured and recorded continuously with a PowerLab system (Powerlab C, 26 series and T series, ADInstruments, Dunedin, New Zealand).

Arterial and mixed venous blood gases were collected via an arterial catheter and the distal port of the Swan–Ganz catheter, respectively. However, blood gas and respiratory data are not included in the present study and will be reported in a subsequent manuscript focusing on respiration and oxygen delivery.

Electrocardiogram (ECG) pads were attached to shaved areas proximal to the carpal joints and one on the sacral region. The three-lead ECG was recorded for HR evaluation and detailed analysis using the PowerLab system.

Cardiac output and body temperature (BT) were measured using the Swan-Ganz pulmonary arterial catheter connected to the Vigilance II continuous cardiac output monitor (Edwards Life Sciences, Irvine, CA, USA). Subsequently, stroke volume (SV) was calculated dividing CO by HR as monitored from the ECG.

To measure fR, a custom 3D-printed facemask, fitted with an airtight silicone seal around the animal’s mouth and nostrils was connected to a spirometry system (Respironics NICO_2_, Respironic Inc. Murrysville, PE, USA). The sheep were trained to breathe via the facemask over a period of three months. The facemask was required to enable spirometry and volumetric capnography; the corresponding ventilation and respiratory function data will be published separately.

After instrumentation was completed, flumazenil 0.01 mg·kg^−1^ (Flumazenil Kabi 0.1 mg·ml^−1^, Fresenius Kabi Austria GmbH, Graz, Austria) was injected IV to antagonise midazolam. The experimental procedure was started 45 min after the administration of flumazenil to allow for washout of these drugs [24, 25].

### Experimental procedure

This randomised, controlled, prospective crossover trial was part of a larger study investigating the potential of two novel drugs to mitigate the hypoxia caused by etorphine. All animals were immobilised three times with etorphine 0.05 mg·kg^−1^ (Captivon; Wildlife Pharmaceuticals, White River, South Africa) intramuscularly (IM) injected in the *quadriceps femoris* muscle. Each sheep was randomly assigned (randomizer.org) to receive one of three treatments: BIMU-8 (1.5 mg·kg^−1^, magistral preparation, BOKU University, Vienna, Austria), vatinoxan (0.15 mg·kg^−1^, Vetcare Ltd., Salo, Finland), or sterile water for injection (control), with a four-week washout period between treatments. All treatments were drawn up in equal volumes (0.08 mL·kg⁻^1^).

Data collection was divided into four phases: baseline (BL), post-etorphine (PE), post-treatment (PT), and post-naltrexone (PN). The variables monitored included fR, systolic, diastolic and mean PAP (sPAP, dPAP, mPAP), CVP, systolic, diastolic and mean AP (sAP, dAP, mAP), ECG, HR, CO, and BT. Although arterial and mixed venous blood gases, spirometry, and additional respiratory parameters were collected concurrently, these data are not presented here and will be reported separately. Monitoring began five minutes before etorphine immobilisation and BL data were recorded over a one-minute period starting four minutes before etorphine injection. Six minutes after immobilisation, all variables were recorded again over one minute (PE). Thereafter, animals received their assigned treatment. All treatments were administered IV over one minute using the introducer sheath into the jugular vein, by a non-blinded author, considered as timepoint zero (T0). During the PT phase, data were collected over a one-minute period at three timepoints with six-minute intervals (T6, T12, T18). Naltrexone (1 mg·kg^−1^; Trexonil, Wildlife Pharmaceuticals Pty Ltd., South Africa) was injected IV 19 minutes after treatment (T19), and all variables were recorded again over one minute, five minutes after reversal (PN). At the end of the procedure, all monitoring equipment was removed, and meloxicam (0.5 mg·kg^−1^; Metacam^®^, Boehringer Ingelheim Pharma GmbH & Co. KG, Ingelheim am Rhein, Germany) was delivered IM.

The sheep were monitored until fully awake and then returned to their stable. The animals were kept on site for at least three days and their behaviour was monitored daily. At the end of each trial, the animals were returned onto their pasture at the Research Institute of Wildlife Ecology. The timeline of the experimental procedure is summarised in Fig. 1.

Predefined termination criteria were established to ensure animal welfare and to terminate the experiment by reversing the immobilisation. These criteria included: (1) mPAP above 50 mmHg, (2) persisting apnoea for longer than 30 s, and (3) arterial-mixed venous oxygen partial pressure gradient falling below 5 mmHg. If the animals exhibited two of the three criteria, the experiment was terminated by administration of naltrexone 1 mg·kg^−1^ IV for this treatment episode. Data collected up to study termination were retained for analysis.

### Data analysis

Statistical analyses were performed in R, version 4.2.2 (R Core Team, 2022). Linear mixed-effects models (lmer function within lme4 R package version 1.1.35.3 [30]) were applied to the physiological variables including fR, dPAP, mPAP, sPAP, dAP, mAP, sAP, CVP, HR, CO, SV, and BT, while ECG data were reported descriptively. Full model results for each physiological variable are provided in the supplementary material.

The initial models included ‘treatment’ (BIMU-8, Vatinoxan, control), ‘time’ (BL, PE, PT, PN), and their interaction as fixed effects. In the PT phase, three time points (T6, T12, and T18) were pooled and analysed as ‘PT’ in the model. Sheep ID was included as a random effect to account for repeated measures. The interaction treatment*time did not improve the models as indicated by the AICc values, and no significance was found. Therefore, the interaction terms were removed, with BT as the only exception. The final models included only ‘treatment’ and ‘time’ as fixed effects for the primary variables, except in the case of BT.

Model diagnostics involved checks for dispersion and evaluation of simulated residuals (*DHARMa*,* v.0.4.6* [26]) for normality, heteroscedasticity, and overall distribution. Autocorrelation analysis was also performed to assess residual independence. Residuals for CVP were not normally distributed and were therefore transformed using Box-Cox transformations. Model fit was re-evaluated following transformation to confirm assumption validity.

The significance of fixed effects was determined using F-tests from the regression model. These tests evaluated the effect of each predictor while accounting for the estimated denominator degrees of freedom (Satterthwaite approximation). Significance threshold was set at *p* *≤* 0.05. The differences within the treatment and time points were tested with post-hoc pairwise comparisons using estimated marginal means (*emmeans*,* v. 1.8.9* [27]), with Tukey’s adjustment applied within each model to control for multiple testing.

## Results

### General results

Six sheep, with an average body weight of 92.2± 9.0 kg, were included in the analysis. Initial signs of etorphine effects, including nystagmus, excitement, and muscle stiffness were observed within 3.7 ± 1.9 min after IM administration. All sheep showed loss of voluntary movement, consistent with adequate immobilisation.

**Fig. 2:**
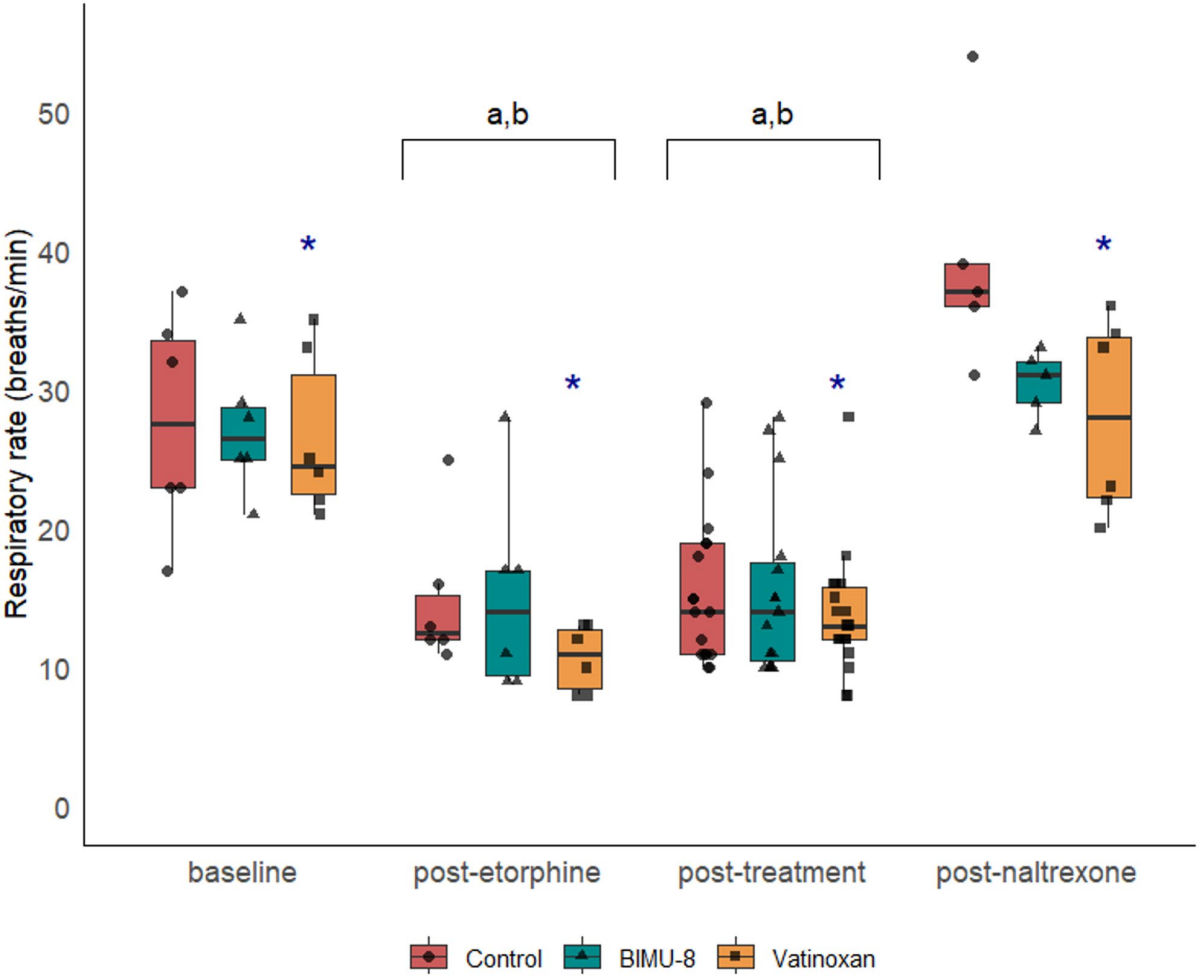
Effects of treatment on respiratory rate. Respiratory rate at four phases: baseline, post-etorphine, post-treatment, and post-naltrexone, in etorphine-immobilised sheep (etorphine 0.05 mg·kg^−1^, *n* = 6) receiving either sterile water (control), BIMU-8 (1.5 mg·kg^−1^), or vatinoxan (0.15 mg·kg^−1^). Values are presented as box plots with individual data points overlaid (● for control, ▲ for BIMU-8, ■ for vatinoxan). The box plots represent the median (horizontal line), interquartile range (IQR), and whiskers extending to 1.5 × IQR. (**a**) *p* < 0.001, significantly different from baseline, (**b**) *p* < 0.001, significantly different from post-naltrexone, (*) *p* < 0.01, significantly different from control (ANOVA type III from the regression model, post hoc emmeans, pairwise contrast for time and treatment)

**Fig. 3:**
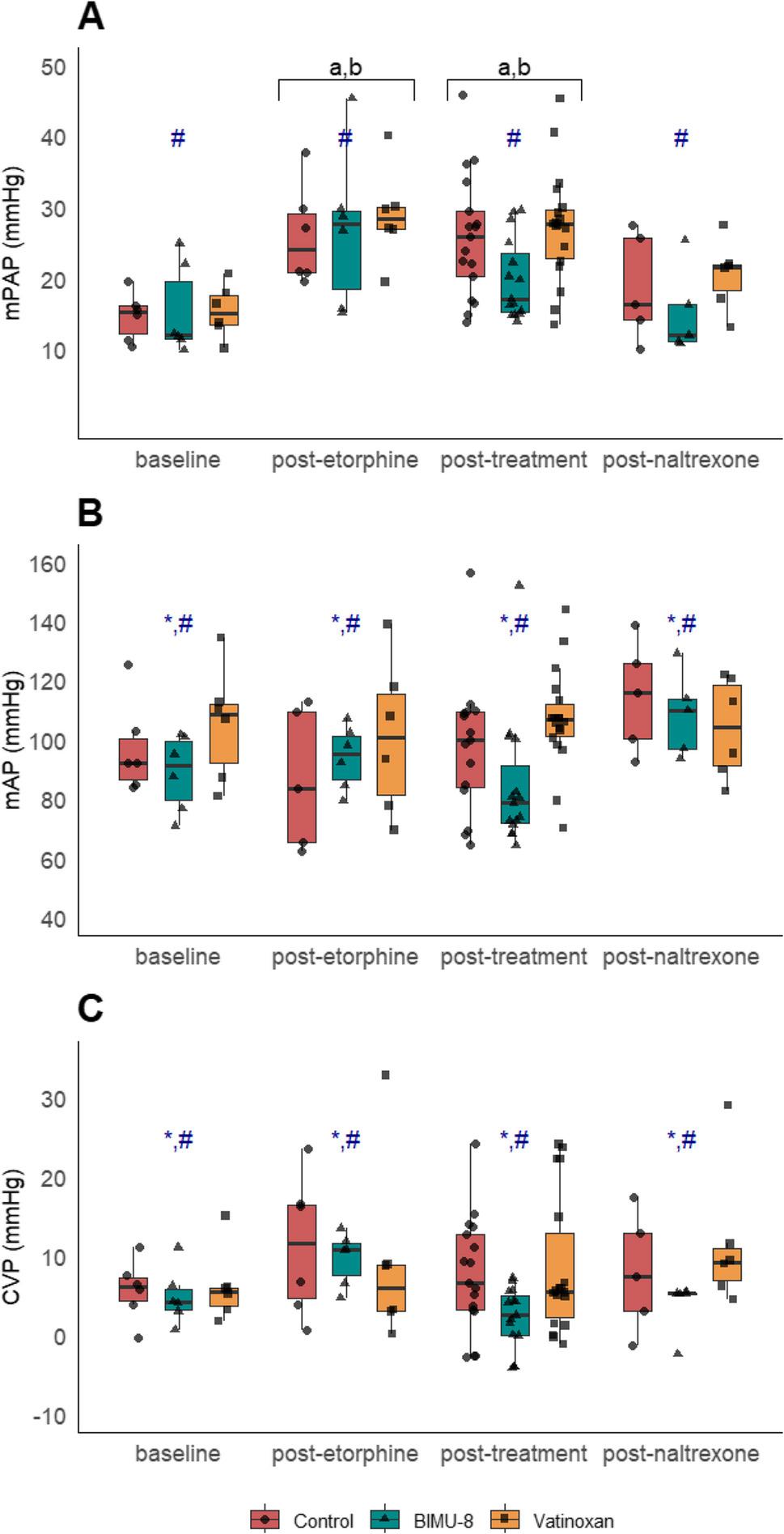
Effects of treatment on mean pulmonary artery pressure (**A**), systolic arterial pressure (**B**), and central venous pressure (**C**). All pressures are presented in four phases: baseline, post-etorphine, post-treatment, and post-naltrexone, in etorphine-immobilised sheep (*n* = 6) receiving either sterile water (control), BIMU-8, or vatinoxan. Values are presented as box plots with individual data points overlaid (● for Control, ▲ for BIMU-8, ■ for vatinoxan). The box plots represent the median (horizontal line), interquartile range (IQR), and whiskers extending to 1.5 × IQR. (**a**) *p* < 0.001, significantly different from baseline. (**b**) *p* < 0.001, significantly different from post-naltrexone. (*). *p* < 0.05, significantly different from control (**#**) *p* < 0.05, significant difference BIMU-8 vs. vatinoxan. BIMU-8 led to significantly lower mPAP, mAP, and CVP

Based on the predefined termination criteria, two animals were antagonised with IV naltrexone before completing the data collection timeline during the third immobilisation session. Both animals recovered uneventful after receiving naltrexone. Consequently, data collected after termination of the experiment were excluded from the analysis from T18 in one sheep of the control group and from T0 in one sheep of the BIMU-8 group. One control animal developed acute right heart failure due to a later diagnosed pre-existing condition immediately after etorphine administration during the initial immobilisation; data was excluded and the animal was replaced with a reserve animal. All variables recorded during the procedures are presented in Figs. 2, 3, 6, and 7 , with a descriptive table (Table 1, S2) and detailed model results (S3) in the supplementary materials.

**Fig. 4:**
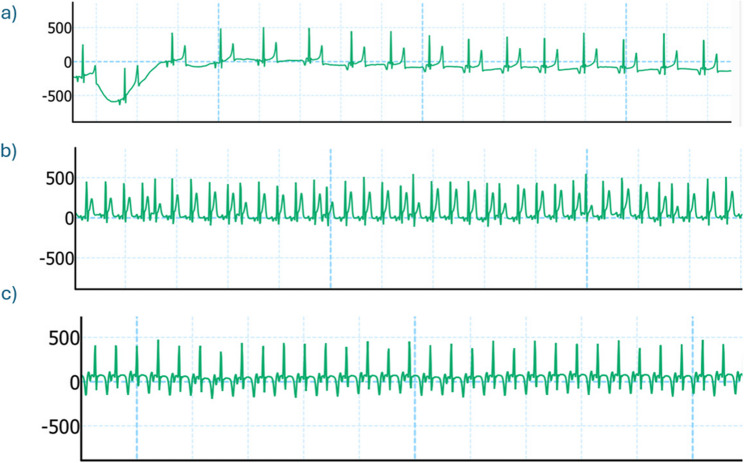
Electrocardiogram showing dysrhythmias after etorphine. **a **Example of normal ECG record during baseline (BL) **b** Example of premature ventricular contractions developed after etorphine administration (PE) **c** Example of bigeminy developed after etorphine administration (PE)

**Fig. 5:**
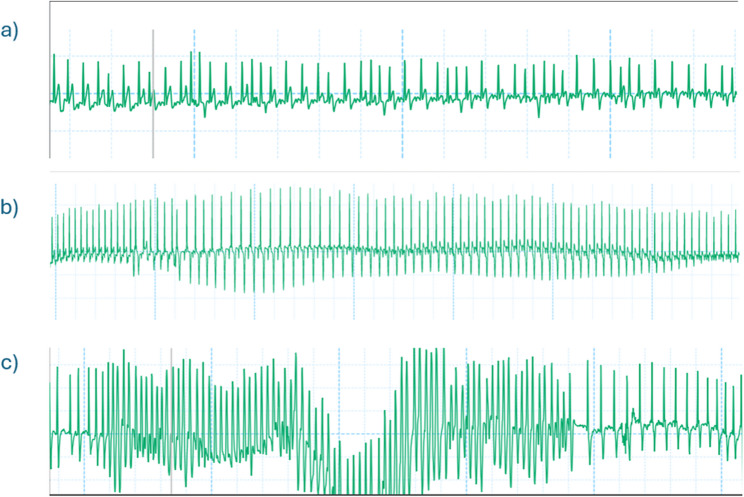
Dysrhythmias recorded within 1–2 minutes after BIMU-8 administration in three different sheep. **a** Irregular rhythm with ventricular tachycardia; **b** Ventricular tachycardia; **c **Ventricular fibrillation followed by Torsades de Pointes (TdP)

**Fig. 6:**
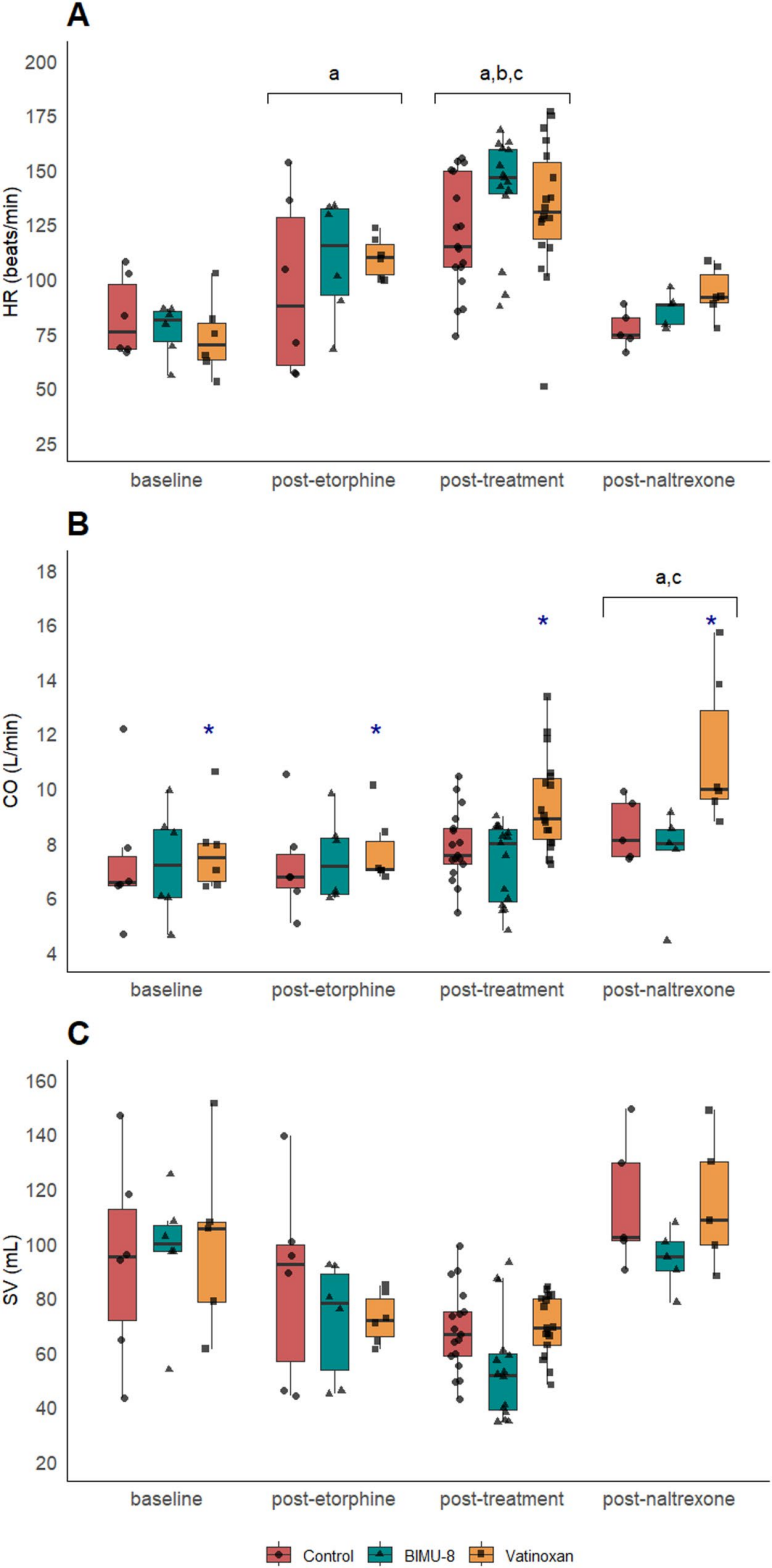
Effects of treatment on heart rate (**A**), cardiac output (**B**), and stroke volume (**C**). All variables are presented in four phases: baseline, post-etorphine, post-treatment, and post-naltrexone, in etorphine-immobilised sheep (*n* = 6) receiving either sterile water (control), BIMU-8, or vatinoxan. Values are presented as box plots with individual data points overlaid (● for Control, ▲ for BIMU-8, ■ for vatinoxan). The box plots represent the median (horizontal line), interquartile range (IQR), and whiskers extending to 1.5 × IQR. (**a**) *p* < 0.005, significantly different from baseline. (**b**) *p* < 0.001, significantly different from post-naltrexone (**c**) *p* < 0.001, significantly different from post-etorphine (*) *p* < 0.05, significantly different from control (**#**) *p* < 0.05, significant difference BIMU-8 vs. vatinoxan. (ANOVA type II, post hoc emmeans, pairwise contrast for time or treatment)

### Respiratory rate

Time had a significant effect on fR (F_3,91_ = 66.23, *p* < 0.001). Following etorphine administration, fR significantly decreased from 27 ± 7 breaths·min^−1^ at BL to 14 ± 5 breaths·min^−1^ at PE and remained lower than BL in all treatments throughout immobilisation (Fig. 2). Respiratory rate returned to baseline levels in all treatments after naltrexone administration. Neither BIMU-8 nor vatinoxan improved fR during immobilisation compared to control. In addition, treatment with vatinoxan had a significant effect (F_2,91_ = 4.13, *p* = 0.019) showing a lower fR compared to the control (*p* = 0.02).

### Pulmonary arterial pressures

Mean pulmonary arterial pressure changed significantly over time (F_3,91_ = 13.46, *p* < 0.001). Etorphine administration induced pulmonary hypertension, with mPAP increasing from 15.2 ± 4.4 mmHg at BL to 27.3 ± 8.0 mmHg at PE and remained significantly higher throughout the immobilisation periods in all treatments (Fig. 3A). Following naltrexone administration, mPAP returned to BL levels in all treatments. Treatment also had a significant effect on mPAP (F_2,92_ = 4.17, *p* = 0.018), with BIMU-8 resulting in significantly lower mPAP compared to animals that received vatinoxan (*p* = 0.02), but not lower compared to control. In the BIMU-8 treatment, values increased from 15.4 ± 6.4 mmHg at BL to 26.9 ± 11.1 mmHg at PE, before decreasing to 19.9 ± 5.6 mmHg after treatment. In contrast, mPAP post-treatment remained elevated in control (26 ± 8.6 mmHg) and vatinoxan (27.4 ± 7.8 mmHg). The same observation was made for both sPAP and dPAP (supplementary materials, S4).

### Arterial pressures

Mean arterial pressure did not change significantly over time. However, individual variability in response to etorphine was observed, with some sheep developing systemic hypertension (mAP > 100 mmHg), while others remained at BL levels (Fig. 3B). Treatment had a significant effect on mAP (F_2,89_= 6.01, *p* = 0.005), with BIMU-8 reducing mAP compared to both the control (*p* = 0.01) and vatinoxan-treated sheep (*p* = 0.01). After BIMU-8 administration, mAP decreased to 84.6 ± 22.3 mmHg at PT, whereas values in the control (106.2 ± 32.0 mmHg) and vatinoxan (106.9 ± 16.9 mmHg) remained comparable to BL and PE. A same observation was made for both sAP and dAP (supplementary materials, S4).

### Central venous pressure

Etorphine induced a slight increase in CVP, though no statistically significant changes were observed over time (Fig. 3C). Baseline CVP was 5.6 ± 3.8 mmHg, increasing to 10.2 ± 8.3 mmHg at PE. Treatment had a significant effect (F_2,91_ = 5.58, *p* = 0.005), with CVP being lower in BIMU-8-treated animals (2.1 ± 3.9 mmHg at PT) compared to those in the control (*p* = 0.02) and in the vatinoxan treatment (*p* = 0.01).

### Electrocardiogram

Twelve out of 18 etorphine immobilisations showed ECG abnormalities six minutes after etorphine administration, including 8 immobilisations where the sheep developed bigeminy and 4 where they showed premature ventricular contractions (Fig. 4). 50% of the sheep (3/6) that received BIMU-8 developed dysrhythmias within 1–2 min post-administration (Fig. 5). In two of these animals, the dysrhythmia resolved within a few minutes, returning to the post-etorphine pattern. However, one animal that developed torsades de pointes (TDP) immediately after receiving BIMU-8 (T0), required reversal of immobilisation before completing the timeline. There was no difference in ECG patterns between PE and PT in vatinoxan-treated sheep.

### Heart rate, cardiac output and stroke volume

Time had a significant effect on HR (F_3,91_ = 33.38, *p* < 0.001) which increased significantly from 77 ± 16 beats·min^−1^ at BL to 105 ± 28 beats·min^−1^ following etorphine administration (*p* < 0.01) and continued to increase during PT in all treatments (*p* < 0.01, BIMU-8: 140 ± 26 beats·min^−1^; control: 120 ± 26 beats·min^−1^; vatinoxan: 133 ± 31 beats·min^−1^) (Fig. 6A). After naltrexone was administered, HR returned to baseline levels.

The effect of time on CO was statistically significant (F_3,91_ = 4.9, *p* = 0.003). CO was higher following naltrexone (9.2 ± 2.6 L·min^−1^) compared to both BL (7.5 ± 2.0 L·min^−1^) and PE (7.4 ± 1.5 L·min^−1^) (Fig. 6B). Treatment itself also had an effect on CO (F_2,91_= 10.88, *p* < 0.001). When the animals received vatinoxan they had significantly higher CO compared to control (*p* < 0.01) and BIMU-8 (*p* < 0.01). During PT, in the BIMU-8, vatinoxan, and control groups CO was 7.3 ± 1.5, 9.4 ± 1.7, and 7.9 ± 1.3 L·min^−1^, respectively.

While CO only increased after naltrexone, SV decreased over time (F_3,92_=17.4, *p* < 0.001) during immobilisation. After etorphine administration, SV decreased from 101.2 ± 33.9 mL at BL to 76.7 ± 24.3 mL at PE and returned to baseline levels only after naltrexone administration (Fig. 6C). Additionally, treatment also had a significant effect on SV (F_2,92_ = 4.4, *p* = 0.014), with SV in BIMU-8-treated sheep was lower compared to vatinoxan (*p* = 0.01).

### Body temperature

**Fig. 7:**
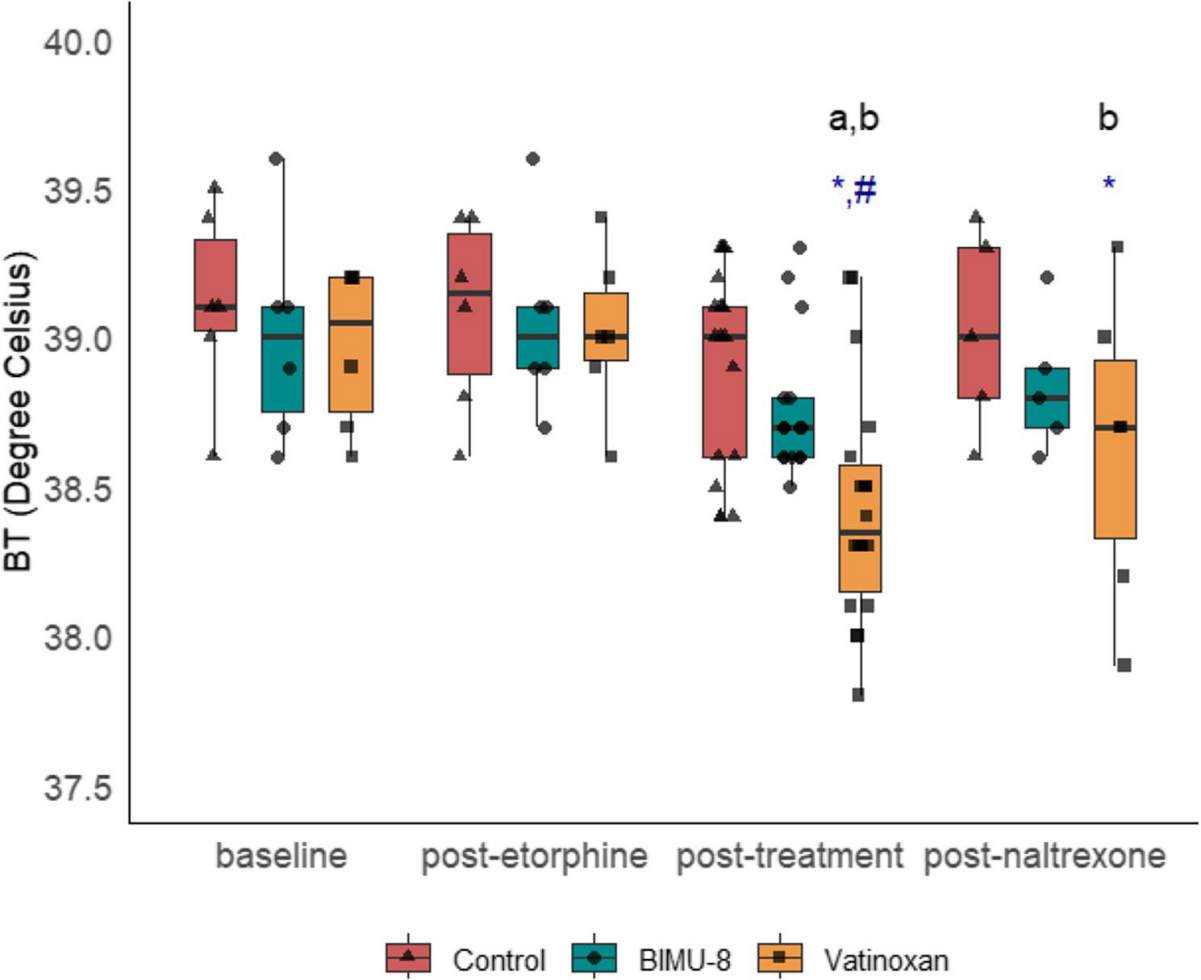
Effects of treatment on body temperature. Body temperature presented at four phases: baseline, post-etorphine, post-treatment, and post-naltrexone, in etorphine-immobilised sheep (*n* = 6) receiving either sterile water (control), BIMU-8, or vatinoxan. Values are presented as box plots with individual data points overlaid (● for Control, ▲ for BIMU-8, ■ for vatinoxan). The box plots represent the median (horizontal line), interquartile range (IQR), and whiskers extending to 1.5 × IQR. (**a**) *p* < 0.001, significantly different from baseline within treatment, (**b**) *p* < 0.001, significantly different from post-etorphine within treatment (*) *p* < 0.02, significantly different control (**#**) *p* < 0.05, significant difference BIMU-8 vs. vatinoxan. (ANOVA type III, post hoc emmeans, pairwise contrast for time and treatment)

A significant interaction between treatment and time was found for vatinoxan during PT (F_6,85_ = 2.2, *p* = 0.049). In contrast to the stable body temperature (BT) observed in the control (38.9 ± 0.3 °C) and BIMU-8 treatment (38.8 ± 0.2 °C), BT was significantly lower in vatinoxan-treated animals during PT (38.4 ± 0.4 °C). Accordingly, BT at BL (38.4 ± 0.4 °C) was significantly higher compared to PT within the vatinoxan-treated animals (*p* < 0.001) (Fig. 7).

## Discussion

Etorphine produced the expected effects in sheep, including bradypnoea, pulmonary hypertension, tachycardia, and a reduction in SV. However, mAP, CVP and CO remained comparable to baseline in all treatments. Treatment with BIMU-8 had no effect on fR but reduced mPAP, mAP, and CVP, and was associated with dysrhythmias. In contrast, animals treated with vatinoxan had lower fR and BT, and higher CO compared to the control, while mPAP, mAP and HR remained unaffected.

### Respiratory compromise

Consistent with previous studies, etorphine induced severe respiratory depression [4, 5, 28, 29]. However, neither BIMU-8 nor vatinoxan improved the fR compared to the control treatment. The lack of effect of BIMU-8 was unexpected, as 5-HT4 receptor activation at the pre-Bötzinger complex has previously been shown to enhance respiratory drive in etorphine-immobilised goats [13]. As the dose of BIMU-8 used in this study was halved compared to the previous study, a dose-dependent effect cannot be excluded. However, due to the occurrence of dysrhythmias in sheep, a higher dose may further compromise cardiovascular function and animal safety. With vatinoxan treatment, fR was lower than in controls. In sheep sedated with xylazine, vatinoxan has previously been shown to abolish the associated increase in fR and dyspnoea while increasing peripheral capillary oxygen saturation [30]. Similarly, in horses anaesthetised with medetomidine, vatinoxan reduced fR and improved tissue perfusion [31]. These findings suggest that, by enhancing oxygenation and tissue perfusion, vatinoxan may reduce the need for compensatory hyperventilation. However, the reduction in fR observed in our study may not be clinically relevant, as several studies have reported no effect of vatinoxan on this parameter [19, 21, 22].

### Vascular compromise

Etorphine has been reported to cause pulmonary and systemic hypertension, potentially mediated by increased peripheral vascular resistance, sympathetic upregulation or hypoxic pulmonary vasoconstriction in different species [4, 9, 14, 15]. In the present study, etorphine consistently induced pulmonary hypertension, in agreement with previous reports, while overall systemic arterial pressure did not change significantly from baseline. BIMU-8 led to a decrease in mPAP, mAP and CVP, without affecting CO. Although BIMU-8 did not result in a statistically detectable reduction in pulmonary hypertension relative to controls, pulmonary hypertension was no longer clinically present (mPAP < 20 mmHg) following treatment. This suggests a vasodilatory effect, most likely mediated through direct 5-HT4 receptor activation and consequent reduction in vascular resistance. In sheep, 5-HT4 receptors have been identified in the pulmonary veins, where their activation induces strong endothelium-independent relaxation [32]. Similar effects of BIMU-8 on blood pressure, and a potential decrease in PAP leading to an improved A–a gradient were observed in etorphine-immobilised goats [13].

In contrast, vatinoxan did not alter mPAP, mAP, and CVP, suggesting a limited effect on pulmonary and systemic vascular pressures in etorphine-immobilised sheep. These results differ from findings in wild boars anaesthetised with medetomidine-tiletamine-zolazepam, where vatinoxan significantly attenuated both pulmonary and systemic hypertension [21]. The observed increase in CO without significant changes in blood pressure in our study indicates that vatinoxan may have induced vasodilation and a reduction in vascular resistance, but not to a degree sufficient to counteract etorphine-induced pulmonary hypertension. Etorphine-induced pulmonary hypertension is likely mediated by multiple mechanisms. Therefore, blocking alpha-2 adrenoceptors in the pulmonary vasculature alone may be insufficient to reduce pulmonary hypertension in etorphine-immobilised sheep. Persistent hypoxaemia may play a major role, as hypoxic pulmonary vasoconstriction can substantially increase pulmonary arterial pressure and counteract the beneficial effects of alpha-2 receptor antagonism. Thus, further investigation into these conditions may help explain the limited effectiveness of vatinoxan in reversing pulmonary hypertension.

Two animals were terminated early during immobilisation based on predefined termination criteria, but recovered uneventfully following intravenous naltrexone administration. One control animal, however, showed acute right-sided heart failure, caused by a previously undetected subclinical pneumonia and precipitated by severe pulmonary hypertension after etorphine administration during the first immobilisation. These observations underline that etorphine can cause significant cardiovascular compromise, which may become rapidly life-threatening in animals with underlying conditions, even if they appear clinically healthy. This underscores the importance of careful monitoring and strict adherence to predefined termination criteria for etorphine immobilisation.

### Cardiac compromise

In our study, etorphine induced dysrhythmias and tachycardia. ECG abnormalities, such as bigeminy and premature ventricular contractions, were observed, similar to those reported in etorphine-immobilised goats [14]. These abnormalities may involve mu-opioid receptors located on cardiac nerves or the vascular endothelium, contributing to reduced ventricular contractility and SV [33, 34]. Moreover, opioids may also affect cardiac myocytes independent of receptor interaction, by blocking ion channels and altering repolarisation or depolarisation [34]. The tachycardia observed is likely multifactorial, as findings in sheep immobilised with etorphine and azaperone suggest that it may arise from hypoxia or hypercapnia, as well as from the baroreceptor reflex activation [15].

BIMU-8 further exacerbated tachycardia and dysrhythmias in sheep, potentially due to its chronotropic and pro-arrhythmic effects identified in other species [35]. In pigs, where, similarly to humans, 5-HT4 receptors are expressed in the atria, heart rate increases through activation of a pathway involving G-proteins in the sinus node [35]. Conversely, in rabbits, which lack 5-HT4 receptors in the heart, BIMU-8 has been shown to inhibit cardiac ion channels, leading to prolonged QT intervals and altered blood flow [36]. Since cardiac 5-HT4 receptor expression and distribution are currently unknown in sheep, the observed dysrhythmias may result from receptor activation, ion channel blockade, or a combination of both. These changes likely contributed to reduced SV, nevertheless CO remained comparable to the control due to the compensatory increase in HR. Although BIMU-8 demonstrated potential beneficial effects, by reducing pulmonary pressures, its clinical application is limited by safety concerns associated with the observed severe dysrhythmias.

In contrast, vatinoxan improved CO, most likely by increasing HR and SV, suggesting a positive inotropic effect and decreasing systemic vascular resistance similar to previous studies in sheep, horses, and dogs sedated with various alpha-2 adrenoceptor agonists [31, 37, 38]. In etorphine-immobilised animals, this action is thought to result from the blockade of alpha-2 adrenoceptors, which inhibit sympathetic tone mediated by circulating endogenous catecholamines [39].

### Effect on body temperature

Vatinoxan decreased BT compared to BIMU-8 or the control. However, its effects on thermoregulation have been inconsistent across species. In beagles sedated with medetomidine and butorphanol, vatinoxan increased superficial temperatures but decreased rectal temperature, suggesting greater peripheral heat loss caused by cutaneous vasodilation [40]. Similarly, in red deer anaesthetised with medetomidine-tiletamine-zolazepam, vatinoxan decreased BT compared to the control group [22]. In contrast, no significant changes were observed in sheep or wild boars anaesthetised with medetomidine combined with different dissociative agents [21, 37]. These findings suggest that vatinoxan’s effects on body temperature are likely influenced by species-specific factors including hair coat, environmental temperature, and anaesthetic protocols. Importantly, reducing BT may be beneficial in etorphine-immobilised animals, which are prone to developing hyperthermia [41, 42].

### Strength, limitations and future studies

This study used sheep as a model for wild ungulates, allowing a comprehensive evaluation of etorphine’s effects and of the potential of adjunct drugs to mitigate its adverse effects. Sheep showed high sensitivity to etorphine, exhibiting the expected cardiovascular and respiratory effects. However, their responses to BIMU-8 differed from those reported in goats and vatinoxan produced only limited effects. These findings support their suitability as a model for evaluating species-specific responses and highlight the importance of species selection when extrapolating results to wild ungulates.

Although BIMU-8 and vatinoxan provided certain cardiovascular benefits, including alleviation of pulmonary hypertension and improved tissue perfusion, further investigation is warranted. Notably, BIMU-8 was associated with severe dysrhythmias, representing a major limitation of its use. Consequently, future approaches combining BIMU-8 with etorphine may be constrained, as they would likely require higher doses and could compromise cardiovascular safety.

Dedicated analyses focusing on respiratory function are furthermore necessary to fully elucidate the effects of BIMU-8 and vatinoxan on oxygenation and hypoxia in etorphine-immobilised animals. Respiratory function, including blood gas analyses and respiratory variables, was assessed in this study but could not be presented within the scope of the current manuscript and will be reported separately.

Since stress may contribute to cardiovascular responses and sympathetic upregulation in immobilised animals, in addition to etorphine’s direct effects, further investigations such as catecholamine measurements are necessary to quantify stress levels. These data could clarify the mechanism of etorphine-induced sympathetic upregulation and the potential of vatinoxan to counteract it. Moreover, although vatinoxan was initially developed as a complementary drug for alpha-2 adrenoceptor agonists, its direct effects remain largely unexplored in non-anaesthetised animals or under different anaesthetic conditions. Studying these effects would provide clearer insight into its pharmacological actions without the confounding influence of alpha-2 agonists and could reveal potential therapeutic applications.

## Conclusion

BIMU-8 provided limited benefits, including modest reductions in pulmonary arterial and systemic blood pressures, but was associated with cardiac risks, notably dysrhythmias. In contrast, vatinoxan increased CO and may have improved peripheral tissue perfusion, although it did not alleviate etorphine-induced pulmonary hypertension. This study highlights the complexity of etorphine-induced cardiopulmonary compromise and the need for further research into the mechanisms driving pulmonary hypertension and hypoxia in immobilised animals, which is essential for developing safer and more effective adjunctive strategies during etorphine immobilisation.

## Supplementary Information

Supplementary Material 1.

## Data Availability

The datasets used and/or analysed during the current study are available from the corresponding author on reasonable request.

## Abbreviations

5-HT4: 5-Hydroxytryptamine 4 (5-HT4) receptor
A-a gradient: Alveolar-arterial pressure gradient
AP: Arterial pressures
BL: Baseline
BT: Body temperature
CO: Cardiac output
CVP: Central venous blood pressure
dAP: Diastolic arterial blood pressure
dPAP: Diastolic pulmonary artery pressure
ECG: Electrocardiogram
HCN: Hyperpolarisation-activated Cyclic Nucleotide–gated
HR: Heart rate
LRT: Log-likelihood ratio tests
mAP: Mean arterial blood pressure
mPAP: Mean pulmonary artery pressure
PAPs: Pulmonary arterial pressures
PE: Post-etorphine
PN: Post-naltrexone
fR: Respiratory rate
sAP: Systolic arterial blood pressure
sPAP: Systolic pulmonary artery pressure
SV: Stroke volume
