# Supplementary material for: Cardiovascular effects of a selective 5-HT4 agonist and an alpha-2 adrenoceptor antagonist in etorphine immobilised sheep (Ovis aries) - a randomised, prospective, and controlled trial
Source: BMC Vet Res. 2026 Mar 6;22:224. doi: 10.1186/s12917-026-05379-x (PMC13078085; doi:10.1186/s12917-026-05379-x)
Supplement: Supplementary file 1 — Supplementary Material 1. [file 12917_2026_5379_MOESM1_ESM.docx]

**Supplementary materials**

**Supplementary material 1: A Sling**

**
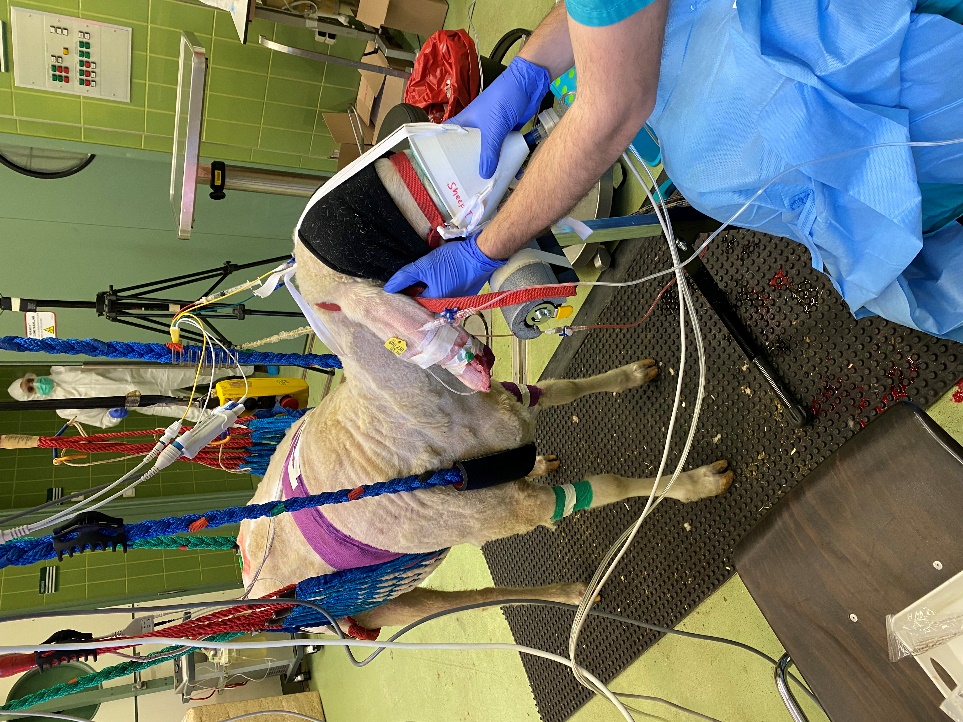

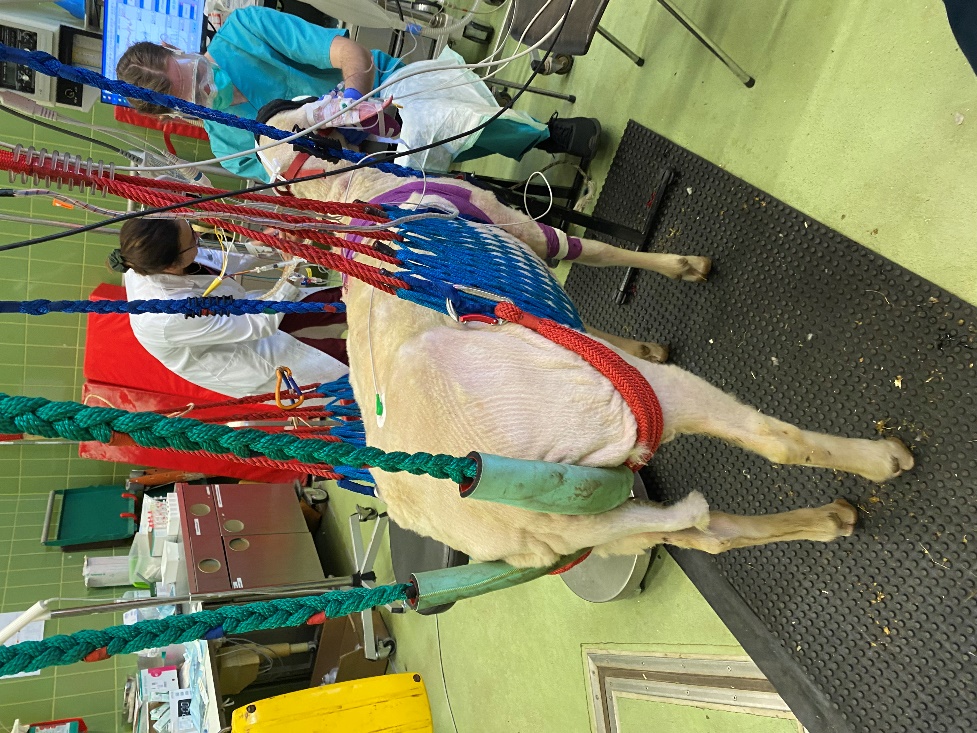
**

Figure 1: A customised sling was used to support the animal’s body weight and maintain a standing position throughout the experiment. It was designed with mesh to support the ventral area and incorporated cushioned ropes that passed through the axillary and inguinal regions. The sling was connected to a ceiling-mounted crane in the equine surgery room and was adjusted according to the height of each sheep.

**Supplementary material 2: Descriptive table**

Table 1: Physiological variables recorded during the procedure are presented in mean ± SD.

| **Variables** | **Treatment** | **Baseline** | **Post-etorphine** | **Post-treatment** | | | **Post-naltrexone** |
| --- | --- | --- | --- | --- | --- | --- | --- |
|  |  |  |  | **T6** | **T12** | **T18** |  |
| **fR (bpm)** | BIMU-8 | 27 ± 5 | 15 ± 7 | 16 ± 8 | 15 ± 7 | 16 ± 6 | 30 ± 2 |
|  | Vatinoxan | 27 ± 6 | 11 ± 2 | 13 ± 2 | 13 ± 3 | 16 ± 7 | 28 ± 7 |
|  | Control | 28 ± 8 | 15 ± 5 | 14 ± 3 | 16 ± 5 | 17 ± 8 | 39 ± 9 |
| **sPAP (mmHg)** | BIMU-8 | 21.9 ± 9.9 | 35.2 ± 13.2 | 26.8 ± 8.0 | 26.0 ± 9.0 | 25.2 ± 9.8 | 21.3 ± 6.9 |
|  | Vatinoxan | 21.9 ± 4.5 | 36.1 ± 6.4 | 33.3 ± 6.5 | 31.1 ± 6.7 | 41.1 ± 19.9 | 28.5 ± 6.8 |
|  | Control | 20.5 ± 3.9 | 34.6 ± 6.5 | 34.0 ± 7.0 | 35.3 ± 11.2 | 30.2 ± 10.3 | 27.2 ± 9.5 |
| **mPAP (mmHg)** | BIMU-8 | 15.4 ± 6.4 | 26.9 ± 11.1 | 20.6 ± 6.0 | 19.8 ± 6.1 | 19.3 ± 6.3 | 15.1 ± 6.1 |
|  | Vatinoxan | 15.4 ± 3.8 | 28.9 ± 6.7 | 27.5 ± 5.7 | 25.2 ± 5.4 | 29.3 ± 11.8 | 20.5 ± 5.0 |
|  | Control | 14.7 ± 3.4 | 26.0 ± 7.0 | 27.2 ± 7.2 | 28.2 ± 11.3 | 21.7 ± 6.4 | 18.8 ± 7.6 |
| **dPAP (mmHg)** | BIMU-8 | 10.2 ± 4.3 | 19.6 ± 10.8 | 14.8 ± 4.4 | 13.9 ± 3.7 | 13.7 ± 3.5 | 9.4 ± 6.7 |
|  | Vatinoxan | 10.5 ± 3.4 | 22.0 ± 7.9 | 21.6 ± 6.1 | 19.3 ± 4.7 | 19.4 ± 6.9 | 12.8 ± 4.6 |
|  | Control | 9.8 ± 3.0 | 19.1 ± 8.8 | 21.1 ± 7.3 | 21.5 ± 10.6 | 14.4 ± 4.1 | 10.6 ± 5.7 |
| **CVP (mmHg)** | BIMU-8 | 4.9 ± 3.5 | 9.7 ± 3.4 | 2.8 ± 4.4 | 1.9 ± 4.3 | 1.7 ± 3.8 | 3.8 ± 3.5 |
|  | Vatinoxan | 6.2 ± 4.7 | 9.5 ± 12.0 | 6.9 ± 8.9 | 6.7 ± 9.0 | 11.9 ± 9.2 | 11.6 ± 8.9 |
|  | Control | 5.7 ± 3.9 | 11.3 ± 8.8 | 7.3 ± 6.8 | 9.5 ± 9.5 | 5.6 ± 5.5 | 7.9 ± 7.5 |
| **sAP (mmHg)** | BIMU-8 | 108.5 ± 20.9 | 116.0 ± 11.9 | 111.1 ± 33.2 | 100.8 ± 18.3 | 97.6 ± 16.9 | 128.3 ± 15.6 |
|  | Vatinoxan | 129.0 ± 21.9 | 125.0 ± 33.5 | 117.2 ± 16.7 | 123.2 ± 26.2 | 129.5 ± 12.9 | 120.3 ± 21.8 |
|  | Control | 118.8 ± 21.3 | 124.5 ± 49.3 | 125.7 ± 41.8 | 126.9 ± 37.0 | 129.0 ± 51.0 | 138.3 ± 22.7 |
| **mAP (mmHg)** | BIMU-8 | 88.8 ± 12.8 | 93.9 ± 10.6 | 94.2 ± 34.9 | 80.3 ± 13.4 | 79.3 ± 12.5 | 108.7 ± 14.3 |
|  | Vatinoxan | 105.4 ± 19.2 | 100.9 ± 26.0 | 102.2 ± 12.9 | 106.0 ± 23.8 | 112.4 ± 13.0 | 103.9 ± 16.7 |
|  | Control | 96.9 ± 15.5 | 100.8 ± 40.4 | 104.7 ± 32.2 | 106.3 ± 29.0 | 108.0 ± 41.8 | 114.7 ± 18.7 |
| **dAP (mmHg)** | BIMU-8 | 77.7 ± 10.1 | 79.4 ± 11.2 | 82.0 ± 36.7 | 67.3 ± 13.1 | 67.3 ± 11.0 | 96.4 ± 14.6 |
|  | Vatinoxan | 92.0 ± 19.0 | 85.6 ± 23.6 | 92.0 ± 12.9 | 94.1 ± 23.0 a | 100.6 ± 13.7 | 92.7 ± 15.6 |
|  | Control | 82.8 ± 9.5 | 84.3 ± 34.8 | 90.0 ± 25.6 | 92.2 ± 24.4 | 94.3 ± 36.1 | 99.1 ± 14.7 |
| **HR (bpm)** | BIMU-8 | 77± 12 | 108 ± 27 | 141 ± 31 | 143 ± 30 | 137 ± 21 | 86 ± 8 |
|  | Vatinoxan | 73 ± 18 | 110 ± 9 | 134 ± 14 | 147 ± 27 | 116 ± 41 | 94 ± 11 |
|  | Control | 83 ± 18 | 96 ± 42 | 126 ± 30 | 122 ± 26 | 110 ± 26 | 77 ± 9 |
| **SV (mL)** | BIMU-8 | 97.2 ± 23.8 | 71.8 ± 21.4 | 53.2 ± 20.1 | 54.9 ± 23.1 | 56.2 ± 20.8 | 94.3 ± 11.1 |
|  | Vatinoxan | 112.5 ± 41.7 | 72.6 ± 9.4 | 63.3 ± 6.4 | 65.5 ± 11.9 | 98.5 ± 41.1 | 125.3 ± 33.7 |
|  | Control | 93.8 ± 36.9 | 85.8 ± 36.1 | 61.8 ± 10.6 | 68.7 ± 18.0 | 75.5 ± 17.0 | 114.5 ± 24.4 |
| **CO (L/min)** | BIMU-8 | 7.25 ± 2.0 | 7.41 ± 1.55 | 7.08 ± 1.27 | 7.34 ± 1.56 | 7.39 ± 1.79 | 7.56 ± 1.84 |
|  | Vatinoxan | 7.73 ± 1.57 | 7.72 ± 1.31 | 8.47 ± 1.2 | 9.49 ± 1.60 | 10.31 ± 1.97 | 11.28 ± 2.78 |
|  | Control | 7.37 ± 2.57 | 7.21 ± 1.86 | 7.58 ± 1.50 | 8.10 ± 1.41 | 8.02 ± 1.12 | 8.48 ± 1.14 |
| **BT(°C)** | BIMU-8 | 39.00 ± 0.36 | 39.05 ± 0.31 | 38.78 ± 0.29 | 38.76 ± 0.21 | 38.76 ± 0.27 | 38.84 ± 0.23 |
|  | Vatinoxan | 38.97 ± 0.27 | 39.02 ± 0.27 | 38.52 ± 0.31 | 38.42 ± 0.43 | 38.37 ± 0.49 | 38.63 ± 0.51 |
|  | Control | 39.12 ± 0.32 | 39.08 ± 0.33 | 38.92 ± 0.38 | 38.88 ± 0.33* | 38.88 ± 0.37 | 39.02 ± 0.33 |

**Supplementary material 3: Model results**

**Respiratory rate**

Model: lmer (RR ~ treatment + time + (1|Sheep.ID), data=data)

Random effects:

| Groups | Name | Variance | Std. Dev. |
| --- | --- | --- | --- |
| Sheep.ID | Intercept | 6.985 | 2.643 |
| Residual | — | 27.322 | 5.227 |

Fixed effects:

|  | Estimate | Std. Error | df | t value | Pr(>\|t\|) | Significance |
| --- | --- | --- | --- | --- | --- | --- |
| (Intercept) | 28.872 | 1.795 | 23.511 | 16.081 | <.0001 | *** |
| treatmentBIMU-8 | -1.534 | 1.299 | 91.857 | -1.181 | 0.24049 |  |
| treatmentVatinoxan | -3.581 | 1.252 | 91.199 | -2.86 | 0.00525 | ** |
| timepost etorphine | -13.611 | 1.742 | 91.087 | -7.812 | <.0001 | *** |
| timepost naltrexone | 5.295 | 1.799 | 91.193 | 2.944 | 0.00412 | ** |
| timepost treatment | -12.254 | 1.439 | 91.2 | -8.516 | <.0001 | *** |

Signif. codes: 0 '***' 0.001 '**' 0.01 '*' 0.05 '.' 0.1 ' ' 1

Type III Analysis of Variance Table with Satterthwaite's method:

| Effect | Sum Sq | Mean Sq | NumDF | DenDF | F value | Pr(>F) | Significance |
| --- | --- | --- | --- | --- | --- | --- | --- |
| treatment | 225.6 | 112.79 | 2 | 91.572 | 4.1282 | 0.01921 | * |
| time | 5428.5 | 1809.52 | 3 | 91.169 | 66.2283 | <.0001 | *** |

Signif. codes: 0 '***' 0.001 '**' 0.01 '*' 0.05 '.' 0.1 ' ' 1

Pairwise comparison of time

| Contrast | Estimate | SE | df | t.ratio | p.value |
| --- | --- | --- | --- | --- | --- |
| baseline - post etorphine | 13.61 | 1.74 | 91 | 7.812 | <.0001 |
| baseline - post naltrexone | -5.29 | 1.8 | 91.1 | -2.943 | 0.0211 |
| baseline - post treatment | 12.25 | 1.44 | 91.1 | 8.513 | <.0001 |
| post etorphine - post naltrexone | -18.91 | 1.8 | 91.1 | -10.508 | <.0001 |
| post etorphine - post treatment | -1.36 | 1.44 | 91.1 | -0.943 | 0.782 |
| post naltrexone - post treatment | 17.55 | 1.5 | 91 | 11.679 | <.0001 |

Results are averaged over the levels of: treatment

Degrees-of-freedom method: kenward-roger

P value adjustment: Tukey method for comparing a family of 4 estimates

Pairwise comparison of treatment

| Contrast | Estimate | SE | df | t.ratio | p.value |
| --- | --- | --- | --- | --- | --- |
| Control - BIMU-8 | 1.53 | 1.3 | 91.8 | 1.179 | 0.4685 |
| Control - Vatinoxan | 3.58 | 1.25 | 91.1 | 2.859 | 0.0144 |
| BIMU-8 - Vatinoxan | 2.05 | 1.28 | 91.6 | 1.598 | 0.2517 |

Results are averaged over the levels of: time

Degrees-of-freedom method: kenward-roger

P value adjustment: tukey method for comparing a family of 3 estimates

**Mean pulmonary arterial pressure**

Model: lmer (mPAP~ treatment + time + (1|Sheep.ID), data=data)

Random effects:

| Group | Name | Variance | Std. Dev. |
| --- | --- | --- | --- |
| Sheep.ID | Intercept | 4.555 | 2.134 |
| Residual |  | 46.157 | 6.794 |

Fixed effects:

| Effect | Estimate | Std. Error | df | t value | p value | Significance |
| --- | --- | --- | --- | --- | --- | --- |
| (Intercept) | 15.743 | 2.058 | 45.984 | 7.649 | <0.0001 | *** |
| treatmentBIMU-8 | -3.185 | 1.684 | 92.551 | -1.891 | 0.0618 | . |
| treatmentVatinoxan | 1.548 | 1.627 | 91.397 | 0.952 | 0.3437 |  |
| timepost etorphine | 12.076 | 2.265 | 91.182 | 5.332 | <0.0001 | *** |
| timepost naltrexone | 3.066 | 2.337 | 91.382 | 1.312 | 0.1929 |  |
| timepost treatment | 9.346 | 1.870 | 91.389 | 4.998 | <0.0001 | *** |

Signif. codes: 0 '***' 0.001 '**' 0.01 '*' 0.05 '.' 0.1 ' ' 1

Type III Analysis of Variance Table with Satterthwaite's method:

| Effect | Sum Sq | Mean Sq | NumDF | DenDF | F value | Pr(>F) | Significance |
| --- | --- | --- | --- | --- | --- | --- | --- |
| treatment | 385.2 | 192.60 | 2 | 92.058 | 4.173 | 0.01842 | * |
| time | 1864.0 | 621.34 | 3 | 91.336 | 13.462 | <.0001 | *** |

Signif. codes: 0 '***' 0.001 '**' 0.01 '*' 0.05 '.' 0.1 ' ' 1

Pairwise comparison of time

| Contrast | Estimate | SE | df | t.ratio | p.value |
| --- | --- | --- | --- | --- | --- |
| baseline - post etorphine | -12.08 | 2.26 | 91.0 | -5.332 | <.0001 |
| baseline - post naltrexone | -3.07 | 2.34 | 91.2 | -1.311 | 0.5583 |
| baseline - post treatment | -9.35 | 1.87 | 91.2 | -4.996 | <.0001 |
| post etorphine - post naltrexone | 9.01 | 2.34 | 91.2 | 3.853 | 0.0012 |
| post etorphine - post treatment | 2.73 | 1.87 | 91.2 | 1.459 | 0.4664 |
| post naltrexone - post treatment | -6.28 | 1.95 | 91.1 | -3.216 | 0.0096 |

Results are averaged over the levels of: treatment

Degrees-of-freedom method: kenward-roger

P value adjustment: Tukey method for comparing a family of 4 estimates

Pairwise comparison of treatment

| Contrast | Estimate | SE | df | t.ratio | p.value |
| --- | --- | --- | --- | --- | --- |
| Control - BIMU-8 | 3.19 | 1.69 | 92.4 | 1.885 | 0.1488 |
| Control - Vatinoxan | -1.55 | 1.63 | 91.2 | -0.951 | 0.6094 |
| BIMU-8 - Vatinoxan | -4.73 | 1.66 | 92.1 | -2.845 | 0.0150 |

Results are averaged over the levels of: time

Degrees-of-freedom method: kenward-roger

P value adjustment: tukey method for comparing a family of 3 estimates

**Systolic pulmonary arterial pressure**

Model: lmer (sPAP~ treatment + time + (1|Sheep.ID), data=data)

Random effects:

| Group | Name | Variance | Std. Dev. |
| --- | --- | --- | --- |
| Sheep.ID | Intercept | 7.063 | 2.658 |
| Residual |  | 78.575 | 8.864 |

Fixed effects:

| Effect | Estimate | Std. Error | df | t value | p value | Significance |
| --- | --- | --- | --- | --- | --- | --- |
| (Intercept) | 22.2340 | 2.6640 | 48.7190 | 8.3470 | <0.0001 | *** |
| treatmentBIMU-8 | -4.0720 | 2.1970 | 92.6620 | -1.8530 | 0.0670 | . |
| treatmentVatinoxan | 1.6650 | 2.1230 | 91.4700 | 0.7840 | 0.4350 |  |
| timepost etorphine | 13.8630 | 2.9550 | 91.2460 | 4.6920 | <0.0001 | *** |
| timepost naltrexone | 4.2450 | 3.0490 | 91.4540 | 1.3920 | 0.1670 |  |
| timepost treatment | 10.1560 | 2.4400 | 91.4610 | 4.1630 | <0.0001 | *** |

Signif. codes: 0 '***' 0.001 '**' 0.01 '*' 0.05 '.' 0.1 ' ' 1

Type III Analysis of Variance Table with Satterthwaite's method:

| Effect | Sum Sq | Mean Sq | NumDF | DenDF | F value | Pr(>F) | Significance |
| --- | --- | --- | --- | --- | --- | --- | --- |
| treatment | 574.58 | 287.29 | 2 | 92.154 | 3.6563 | 0.02964 | * |
| time | 2227.37 | 742.46 | 3 | 91.406 | 9.449 | 0.00002 | *** |

Signif. codes: 0 '***' 0.001 '**' 0.01 '*' 0.05 '.' 0.1 ' ' 1

Pairwise comparison of time

| Contrast | Estimate | SE | df | t.ratio | p.value |
| --- | --- | --- | --- | --- | --- |
| baseline - post etorphine | -13.86 | 2.95 | 91 | -4.692 | 0.0001 |
| baseline - post naltrexone | -4.24 | 3.05 | 91.2 | -1.391 | 0.508 |
| baseline - post treatment | -10.16 | 2.44 | 91.2 | -4.161 | 0.0004 |
| post etorphine - post naltrexone | 9.62 | 3.05 | 91.2 | 3.153 | 0.0116 |
| post etorphine - post treatment | 3.71 | 2.44 | 91.2 | 1.519 | 0.4305 |
| post naltrexone - post treatment | -5.91 | 2.55 | 91.1 | -2.32 | 0.101 |

Results are averaged over the levels of: treatment

Degrees-of-freedom method: kenward-roger

P value adjustment: Tukey method for comparing a family of 4 estimates

Pairwise comparison of treatment

| Contrast | Estimate | SE | df | t.ratio | p.value |
| --- | --- | --- | --- | --- | --- |
| Control - BIMU-8 | 4.07 | 2.2 | 92.5 | 1.847 | 0.1602 |
| Control - Vatinoxan | -1.66 | 2.12 | 91.2 | -0.784 | 0.7139 |
| BIMU-8 - Vatinoxan | -5.74 | 2.17 | 92.1 | -2.643 | 0.0259 |

Results are averaged over the levels of: time

Degrees-of-freedom method: kenward-roger

P value adjustment: tukey method for comparing a family of 3 estimates

**Diastolic pulmonary arterial pressure**

Model: lmer (dPAP~ treatment + time + (1|Sheep.ID), data=data)

Random effects:

| Group | Name | Variance | Std. Dev. |
| --- | --- | --- | --- |
| Sheep.ID | Intercept | 5.592 | 2.365 |
| Residual |  | 34.888 | 5.907 |

Fixed effects:

| Effect | Estimate | Std. Error | df | t value | p value | Significance |
| --- | --- | --- | --- | --- | --- | --- |
| (Intercept) | 10.616 | 1.887 | 32.935 | 5.626 | <0.0001 | *** |
| treatmentBIMU-8 | -2.488 | 1.466 | 92.082 | -1.697 | 0.0931 | . |
| treatmentVatinoxan | 1.23 | 1.415 | 91.168 | 0.869 | 0.3869 |  |
| timepost etorphine | 10.032 | 1.969 | 91.007 | 5.095 | <0.0001 | *** |
| timepost naltrexone | 1.012 | 2.032 | 91.158 | 0.498 | 0.6196 |  |
| timepost treatment | 7.855 | 1.626 | 91.166 | 4.831 | <0.0001 | *** |

Signif. codes: 0 '***' 0.001 '**' 0.01 '*' 0.05 '.' 0.1 ' ' 1

Type III Analysis of Variance Table with Satterthwaite's method:

| Effect | Sum Sq | Mean Sq | NumDF | DenDF | F value | Pr(>F) | Significance |
| --- | --- | --- | --- | --- | --- | --- | --- |
| treatment | 236.82 | 118.41 | 2 | 91.688 | 3.394 | 0.03784 | * |
| time | 1504.86 | 501.62 | 3 | 91.124 | 14.378 | <0.0001 | *** |

Signif. codes: 0 '***' 0.001 '**' 0.01 '*' 0.05 '.' 0.1 ' ' 1

Pairwise comparison of time

| Contrast | Estimate | SE | df | t.ratio | p.value |
| --- | --- | --- | --- | --- | --- |
| baseline - post etorphine | -10.03 | 1.97 | 91 | -5.095 | <.0001 |
| baseline - post naltrexone | -1.01 | 2.03 | 91.2 | -0.498 | 0.9593 |
| baseline - post treatment | -7.85 | 1.63 | 91.2 | -4.829 | <.0001 |
| post etorphine - post naltrexone | 9.02 | 2.03 | 91.2 | 4.436 | 0.0001 |
| post etorphine - post treatment | 2.18 | 1.63 | 91.2 | 1.338 | 0.5411 |
| post naltrexone - post treatment | -6.84 | 1.7 | 91.1 | -4.03 | 0.0007 |

Results are averaged over the levels of: treatment

Degrees-of-freedom method: kenward-roger

P value adjustment: Tukey method for comparing a family of 4 estimates

Pairwise comparison of treatment

| Contrast | Estimate | SE | df | t.ratio | p.value |
| --- | --- | --- | --- | --- | --- |
| Control - BIMU-8 | 2.49 | 1.47 | 92.1 | 1.693 | 0.2134 |
| Control - Vatinoxan | -1.23 | 1.42 | 91.2 | -0.869 | 0.6611 |
| BIMU-8 - Vatinoxan | -3.72 | 1.45 | 91.8 | -2.57 | 0.0313 |

Results are averaged over the levels of: time

Degrees-of-freedom method: kenward-roger

P value adjustment: tukey method for comparing a family of 3 estimates

**Mean arterial pressure**

Model: lmer (mAP ~ treatment + time + (1|Sheep.ID), data=data)

Random effects:

| Group | Effect | Variance | Std. Dev. |
| --- | --- | --- | --- |
| Sheep.ID | Intercept | 105.0 | 10.25 |
| Residual |  | 425.3 | 20.62 |

Fixed effects:

| Fixed Effect | Estimate | Std. Error | Degrees of Freedom | t value | p value | Significance |
| --- | --- | --- | --- | --- | --- | --- |
| (Intercept) | 101.8889 | 7.0396 | 23.9797 | 14.474 | <0.0001 | *** |
| treatmentBIMU-8 | -14.8532 | 5.1235 | 91.8405 | -2.899 | 0.0047 | ** |
| treatmentVatinoxan | 0.2232 | 4.9396 | 91.1605 | 0.045 | 0.9641 |  |
| timepost etorphine | 1.5261 | 6.8742 | 91.0442 | 0.222 | 0.8248 |  |
| timepost naltrexone | 10.7589 | 7.0964 | 91.1537 | 1.516 | 0.1330 |  |
| timepost treatment | 1.9308 | 5.6774 | 91.1607 | 0.34 | 0.7346 |  |

Signif. codes: 0 '***' 0.001 '**' 0.01 '*' 0.05 '.' 0.1 ' ' 1

Type III Analysis of Variance Table with Satterthwaite's method:

| Factor | Sum Sq | Mean Sq | NumDF | DenDF | F value | p value | Significance |
| --- | --- | --- | --- | --- | --- | --- | --- |
| treatment | 4826.8 | 2413.4 | 2 | 91.546 | 5.6748 | 0.00475 | ** |
| time | 1217.3 | 405.76 | 3 | 91.13 | 0.9541 | 0.418 |  |

Signif. codes: 0 '***' 0.001 '**' 0.01 '*' 0.05 '.' 0.1 ' ' 1

Pairwise comparison of time

| Contrast | Estimate | SE | df | t ratio | p value |
| --- | --- | --- | --- | --- | --- |
| baseline - post etorphine | -1.526 | 6.87 | 91 | -0.222 | 0.9961 |
| baseline - post naltrexone | -10.759 | 7.1 | 91.1 | -1.516 | 0.4324 |
| baseline - post treatment | -1.931 | 5.68 | 91.1 | -0.34 | 0.9864 |
| post etorphine - post naltrexone | -9.233 | 7.1 | 91.1 | -1.301 | 0.5649 |
| post etorphine - post treatment | -0.405 | 5.68 | 91.1 | -0.071 | 0.9999 |
| post naltrexone - post treatment | 8.828 | 5.93 | 91 | 1.489 | 0.4482 |

Results are averaged over the levels of: treatment

Degrees-of-freedom method: kenward-roger

P value adjustment: Tukey method for comparing a family of 4 estimates

Pairwise comparison of treatment

| Contrast | Estimate | SE | df | t ratio | p value |
| --- | --- | --- | --- | --- | --- |
| Control - BIMU-8 | 14.853 | 5.13 | 91.8 | 2.894 | 0.0131 |
| Control - Vatinoxan | -0.223 | 4.94 | 91.1 | -0.045 | 0.9989 |
| BIMU-8 - Vatinoxan | -15.076 | 5.05 | 91.6 | -2.984 | 0.0101 |

Results are averaged over the levels of: time

Degrees-of-freedom method: kenward-roger

P value adjustment: tukey method for comparing a family of 3 estimates

**Systolic arterial pressure**

Model: lmer (SAP ~ treatment + time + (1|Sheep.ID), data=data)

Random effects:

| Group | Effect | Variance | Std. Dev. |
| --- | --- | --- | --- |
| Sheep.ID | Intercept | 168.4 | 12.98 |
| Residual |  | 604.5 | 24.59 |

Fixed effects:

| Fixed Effect | Estimate | Std. Error | Degrees of Freedom | t value | p value | Significance |
| --- | --- | --- | --- | --- | --- | --- |
| (Intercept) | 125.295 | 8.581 | 21.990 | 14.602 | <0.001 | *** |
| treatmentBIMU-8 | -17.006 | 6.110 | 91.804 | -2.784 | 0.00653 | ** |
| treatmentVatinoxan | -2.631 | 5.889 | 91.185 | -0.447 | 0.65617 |  |
| timepost etorphine | 3.078 | 8.195 | 91.080 | 0.376 | 0.70813 |  |
| timepost naltrexone | 8.673 | 8.461 | 91.179 | 1.025 | 0.30803 |  |
| timepost treatment | -1.186 | 6.769 | 91.186 | -0.175 | 0.86131 |  |

Signif. codes: 0 '***' 0.001 '**' 0.01 '*' 0.05 '.' 0.1 ' ' 1

Type III Analysis of Variance Table with Satterthwaite's method:

| Factor | Sum Sq | Mean Sq | NumDF | DenDF | F value | p value | Significance |
| --- | --- | --- | --- | --- | --- | --- | --- |
| treatment | 5392.1 | 2696.05 | 2 | 91.54 | 4.4601 | 0.01418 | * |
| time | 1264.4 | 421.48 | 3 | 91.16 | 0.6973 | 0.55609 |  |

Signif. codes: 0 '***' 0.001 '**' 0.01 '*' 0.05 '.' 0.1 ' ' 1

Pairwise comparison of time

| Contrast | Estimate | SE | df | t ratio | p value |
| --- | --- | --- | --- | --- | --- |
| baseline - post etorphine | -3.08 | 8.20 | 91.0 | -0.376 | 0.9818 |
| baseline - post naltrexone | -8.67 | 8.46 | 91.1 | -1.025 | 0.7353 |
| baseline - post treatment | 1.19 | 6.77 | 91.1 | 0.175 | 0.9981 |
| post etorphine - post naltrexone | -5.60 | 8.46 | 91.1 | -0.661 | 0.9113 |
| post etorphine - post treatment | 4.26 | 6.77 | 91.1 | 0.630 | 0.9222 |
| post naltrexone - post treatment | 9.86 | 7.07 | 91.0 | 1.395 | 0.5057 |

Results are averaged over the levels of: treatment

Degrees-of-freedom method: kenward-roger

P value adjustment: Tukey method for comparing a family of 4 estimates

Pairwise comparison of treatment

| Contrast | Estimate | SE | df | t ratio | p value |
| --- | --- | --- | --- | --- | --- |
| Control - BIMU-8 | 17.01 | 6.12 | 91.7 | 2.779 | 0.0180 |
| Control - Vatinoxan | 2.63 | 5.89 | 91.1 | 0.447 | 0.8960 |
| BIMU-8 - Vatinoxan | -14.38 | 6.02 | 91.6 | -2.387 | 0.0495 |

Results are averaged over the levels of: time

Degrees-of-freedom method: kenward-roger

P value adjustment: tukey method for comparing a family of 3 estimates

**Diastolic arterial pressure**

Model: lmer (dAP ~ treatment + time + (1|Sheep.ID), data=data)

Random effects:

| Group | Effect | Variance | Std. Dev. |
| --- | --- | --- | --- |
| Sheep.ID | Intercept | 74.17 | 8.612 |
| Residual |  | 358.75 | 18.941 |

Fixed effects:

| Fixed Effect | Estimate | Std. Error | Degrees of Freedom | t value | p value | Significance |
| --- | --- | --- | --- | --- | --- | --- |
| (Intercept) | 87.555 | 6.277 | 27.193 | 13.949 | <0.0001 | *** |
| treatmentBIMU-8 | -12.765 | 4.704 | 91.895 | -2.714 | 0.00795 | ** |
| treatmentVatinoxan | 2.572 | 4.537 | 91.116 | 0.567 | 0.57215 |  |
| timepost etorphine | -1.057 | 6.314 | 90.981 | -0.167 | 0.86746 |  |
| timepost naltrexone | 10.748 | 6.517 | 91.108 | 1.649 | 0.10258 |  |
| timepost treatment | 2.166 | 5.214 | 91.115 | 0.415 | 0.67883 |  |

Signif. codes: 0 '***' 0.001 '**' 0.01 '*' 0.05 '.' 0.1 ' ' 1

Type III Analysis of Variance Table with Satterthwaite's method:

| Factor | Sum Sq | Mean Sq | NumDF | DenDF | F value | p value | Significance |
| --- | --- | --- | --- | --- | --- | --- | --- |
| treatment | 4396.4 | 2198.18 | 2 | 91.558 | 6.1273 | 0.003181 | ** |
| time | 1429 | 476.32 | 3 | 91.079 | 1.3277 | 0.270268 |  |

Signif. codes: 0 '***' 0.001 '**' 0.01 '*' 0.05 '.' 0.1 ' ' 1

Pairwise comparison of time

| Contrast | Estimate | SE | df | t ratio | p value |
| --- | --- | --- | --- | --- | --- |
| baseline - post etorphine | 1.06 | 6.31 | 91 | 0.167 | 0.9983 |
| baseline - post naltrexone | -10.75 | 6.52 | 91.1 | -1.649 | 0.357 |
| baseline - post treatment | -2.17 | 5.22 | 91.1 | -0.415 | 0.9757 |
| post etorphine - post naltrexone | -11.8 | 6.52 | 91.1 | -1.811 | 0.275 |
| post etorphine - post treatment | -3.22 | 5.22 | 91.1 | -0.618 | 0.9261 |
| post naltrexone - post treatment | 8.58 | 5.44 | 91 | 1.576 | 0.3973 |

Results are averaged over the levels of: treatment

Degrees-of-freedom method: kenward-roger

P value adjustment: Tukey method for comparing a family of 4 estimates

Pairwise comparison of treatment

| Contrast | Estimate | SE | df | t ratio | p value |
| --- | --- | --- | --- | --- | --- |
| Control - BIMU-8 | 12.77 | 4.71 | 91.9 | 2.708 | 0.0218 |
| Control - Vatinoxan | -2.57 | 4.54 | 91.1 | -0.567 | 0.8381 |
| BIMU-8 - Vatinoxan | -15.34 | 4.64 | 91.7 | -3.306 | 0.0038 |

Results are averaged over the levels of: time

Degrees-of-freedom method: kenward-roger

P value adjustment: tukey method for comparing a family of 3 estimates

**Central venous pressure**

Model: lmer (CVP_tr~ treatment + time + (1|Sheep.ID), data=data)

Random effects:

| Group | Effect | Variance | Std. Dev. |
| --- | --- | --- | --- |
| Sheep.ID | **Intercept** | **0.1955** | **0.4421** |
| Residual |  | **0.7489** | **0.8654** |

Fixed effects:

| Effect | Estimate | Std. Error | df | t value | p value | Significance |
| --- | --- | --- | --- | --- | --- | --- |
| (Intercept) | 0.0608 | 0.2984 | 22.7 | 0.204 | 0.8403 |  |
| treatmentBIMU-8 | -0.5891 | 0.2150 | 91.7 | -2.740 | 0.00739 | ** |
| treatmentVatinoxan | 0.0605 | 0.2073 | 91.1 | 0.292 | 0.7709 |  |
| timepost etorphine | 0.5453 | 0.2885 | 91.0 | 1.890 | 0.0619 | . |
| timepost naltrexone | 0.2835 | 0.2978 | 91.1 | 0.952 | 0.3436 |  |
| timepost treatment | -0.0571 | 0.2382 | 91.1 | -0.240 | 0.8110 |  |

Signif. codes: 0 '***' 0.001 '**' 0.01 '*' 0.05 '.' 0.1 ' ' 1

Type III Analysis of Variance Table with Satterthwaite's method:

| Factor | Sum Sq | Mean Sq | NumDF | DenDF | F value | p value | Significance |
| --- | --- | --- | --- | --- | --- | --- | --- |
| treatment | 8.3508 | 4.1754 | 2 | 91.46 | 5.576 | 0.00519 | ** |
| time | 5.4970 | 1.8323 | 3 | 91.05 | 2.447 | 0.06884 . |  |

Signif. codes: 0 '***' 0.001 '**' 0.01 '*' 0.05 '.' 0.1 ' ' 1

Pairwise comparison of time

| Contrast | Estimate | SE | df | t ratio | p value |
| --- | --- | --- | --- | --- | --- |
| baseline - post etorphine | -0.5453 | 0.288 | 91 | -1.890 | 0.2394 |
| baseline - post naltrexone | -0.2835 | 0.298 | 91 | -0.952 | 0.7769 |
| baseline - post treatment | 0.0571 | 0.238 | 91 | 0.240 | 0.9951 |
| post etorphine - post naltrexone | 0.2617 | 0.298 | 91 | 0.879 | 0.8159 |
| post etorphine - post treatment | 0.6024 | 0.238 | 91 | 2.528 | 0.0623 |
| post naltrexone - post treatment | 0.3407 | 0.249 | 91 | 1.369 | 0.5217 |

Results are averaged over the levels of: treatment

Degrees-of-freedom method: kenward-roger

P value adjustment: Tukey method for comparing a family of 4 estimates

Pairwise comparison of treatment

| Contrast | Estimate | SE | df | t ratio | p value |
| --- | --- | --- | --- | --- | --- |
| Control - BIMU-8 | 0.5891 | 0.215 | 92 | 2.735 | 0.0203 |
| Control - Vatinoxan | -0.0605 | 0.207 | 91 | -0.292 | 0.9541 |
| BIMU-8 - Vatinoxan | -0.6496 | 0.212 | 92 | -3.064 | 0.0080 |

Results are averaged over the levels of: time

Degrees-of-freedom method: kenward-roger

P value adjustment: tukey method for comparing a family of 3 estimates

**Heart rate**

Model: lmer (HR ~ treatment + time + (1|Sheep.ID), data=data)

Random effects:

| Group | Name | Variance | Std. Dev. |
| --- | --- | --- | --- |
| Sheep.ID | Intercept | 96.94 | 9.846 |
| Residual | - | 503.83 | 22.446 |

Fixed effects:

|  | Estimate | Std. Error | df | t value | Pr(>\|t\|) | Significances |
| --- | --- | --- | --- | --- | --- | --- |
| (Intercept) | 70.255 | 7.357 | 28.996 | 9.549 | 1.86E-10 | *** |
| treatmentBIMU-8 | 12.386 | 5.574 | 91.996 | 2.222 | 0.028726 | * |
| treatmentVatinoxan | 9.094 | 5.376 | 91.189 | 1.691 | 0.09416 | . |
| timepost etorphine | 27.696 | 7.482 | 91.049 | 3.702 | 0.000367 | *** |
| timepost naltrexone | 9.375 | 7.723 | 91.181 | 1.214 | 0.227951 |  |
| timepost treatment | 53.758 | 6.179 | 91.188 | 8.7 | 1.32E-13 | *** |

Signif. codes: 0 '***' 0.001 '**' 0.01 '*' 0.05 '.' 0.1 ' ' 1

Type III Analysis of Variance Table with Satterthwaite's method:

| Effect | Sum Sq | Mean Sq | Num DF | Den DF | F value | Pr(>F) | Significance |
| --- | --- | --- | --- | --- | --- | --- | --- |
| treatment | 2717 | 1358.6 | 2 | 91.647 | 2.70 | 0.0728 | . |
| time | 50459 | 16819.5 | 3 | 91.151 | 33.38 | 1.19e-14 | *** |

Signif. codes: 0 '***' 0.001 '**' 0.01 '*' 0.05 '.' 0.1 ' ' 1

Pairwise comparison of time

| Contrast | Estimate | SE | df | t.ratio | p.value |
| --- | --- | --- | --- | --- | --- |
| baseline - post etorphine | -27.70 | 7.48 | 91.0 | -3.702 | 0.0020 |
| baseline - post naltrexone | -9.37 | 7.73 | 91.1 | -1.213 | 0.6200 |
| baseline - post treatment | -53.76 | 6.18 | 91.1 | -8.697 | <.0001 |
| post etorphine - post naltrexone | 18.32 | 7.73 | 91.1 | 2.371 | 0.0900 |
| post etorphine - post treatment | -26.06 | 6.18 | 91.1 | -4.217 | 0.0003 |
| post naltrexone - post treatment | -44.38 | 6.45 | 91.1 | -6.879 | <.0001 |

Results are averaged over the levels of: treatment

Degrees-of-freedom method: kenward-roger

P value adjustment: Tukey method for comparing a family of 4 estimates

Pairwise comparison of treatment

| Contrast | Estimate | SE | df | t.ratio | p.value |
| --- | --- | --- | --- | --- | --- |
| Control - BIMU-8 | -12.39 | 5.59 | 92.0 | -2.217 | 0.0736 |
| Control - Vatinoxan | -9.09 | 5.38 | 91.1 | -1.691 | 0.2142 |
| BIMU-8 - Vatinoxan | 3.29 | 5.50 | 91.7 | 0.599 | 0.8211 |

Results are averaged over the levels of: time

Degrees-of-freedom method: kenward-roger

P value adjustment: tukey method for comparing a family of 3 estimates

**Cardiac output**

Model: lmer (CO ~ treatment + time + (1|Sheep.ID), data=data)

Random effects:

| Group | Effect | Variance | Std. Dev. |
| --- | --- | --- | --- |
| Sheep.ID | Intercept | 0.5185 | 0.720 |
| Residual |  | 2.5866 | 1.608 |

Fixed effects:

| Fixed Effect | Estimate | Std. Error | Degrees of Freedom | t value | p value | Significance |
| --- | --- | --- | --- | --- | --- | --- |
| (Intercept) | 7.1655 | 0.5304 | 27.70 | 13.509 | <0.001 | *** |
| treatmentBIMU-8 | -0.4456 | 0.3994 | 91.89 | -1.116 | 0.267 |  |
| treatmentVatinoxan | 1.2991 | 0.3852 | 91.09 | 3.373 | 0.001 | ** |
| timepost etorphine | -0.0067 | 0.5361 | 90.95 | -0.012 | 0.990 |  |
| timepost naltrexone | 1.8198 | 0.5534 | 91.08 | 3.288 | 0.001 | ** |
| timepost treatment | 0.7981 | 0.4427 | 91.09 | 1.803 | 0.075 | . |

Signif. codes: 0 '***' 0.001 '**' 0.01 '*' 0.05 '.' 0.1 ' ' 1

Type III Analysis of Variance Table with Satterthwaite's method:

| Factor | Sum Sq | Mean Sq | NumDF | DenDF | F value | p value | Significance |
| --- | --- | --- | --- | --- | --- | --- | --- |
| treatment | 56.281 | 28.141 | 2 | 91.54 | 10.880 | <0.001 | *** |
| time | 38.117 | 12.706 | 3 | 91.05 | 4.912 | 0.003 | ** |

Signif. codes: 0 '***' 0.001 '**' 0.01 '*' 0.05 '.' 0.1 ' ' 1

Pairwise comparison of time

| Contrast | Estimate | SE | df | t ratio | p value |
| --- | --- | --- | --- | --- | --- |
| baseline - post etorphine | 0.0067 | 0.536 | 91.0 | 0.012 | 1.000 |
| baseline - post naltrexone | -1.8198 | 0.554 | 91.1 | -3.287 | 0.0077 |
| baseline - post treatment | -0.7981 | 0.443 | 91.1 | -1.802 | 0.279 |
| post etorphine - post naltrexone | -1.8265 | 0.554 | 91.1 | -3.299 | 0.0074 |
| post etorphine - post treatment | -0.8048 | 0.443 | 91.1 | -1.817 | 0.272 |
| post naltrexone - post treatment | 1.0217 | 0.462 | 91.0 | 2.210 | 0.128 |

Results are averaged over the levels of: treatment

Degrees-of-freedom method: kenward-roger

P value adjustment: Tukey method for comparing a family of 4 estimates

Pairwise comparison of treatment

| Contrast | Estimate | SE | df | t ratio | p value |
| --- | --- | --- | --- | --- | --- |
| Control - BIMU-8 | 0.446 | 0.400 | 91.9 | 1.113 | 0.508 |
| Control - Vatinoxan | -1.299 | 0.385 | 91.1 | -3.371 | 0.0031 |
| BIMU-8 - Vatinoxan | -1.745 | 0.394 | 91.7 | -4.429 | 0.0001 |

Results are averaged over the levels of: time

Degrees-of-freedom method: kenward-roger

P value adjustment: tukey method for comparing a family of 3 estimates

**Stroke volume**

Model: lmer (SV ~ treatment + time + (1|Sheep.ID), data=data)

Random effects:

| **Group** | **Name** | **Variance** | **Std. Dev.** |
| --- | --- | --- | --- |
| Sheep.ID | (Intercept) | 19.71 | 4.44 |
| Residual |  | 630.42 | 25.11 |

| Fixed effects: | |  |  |  |  |  |
| --- | --- | --- | --- | --- | --- | --- |
| **Fixed Effect** | **Estimate** | **Std. Error** | **df** | **t value** | **Pr(>\|t\|)** | **Significance** |
| **(Intercept)** | 102.929 | 7.123 | 70.535 | 14.451 | < 2e-16 | *** |
| **treatmentBIMU-8** | -11.684 | 6.207 | 93.319 | -1.883 | 0.06287 | . |
| **treatmentVatinoxan** | 6.392 | 6.01 | 91.628 | 1.064 | 0.29029 |  |
| **timepost etorphine** | -24.472 | 8.369 | 91.273 | -2.924 | 0.00436 | ** |
| **timepost naltrexone** | 10.658 | 8.634 | 91.599 | 1.234 | 0.22018 |  |
| **timepost treatment** | -34.832 | 6.907 | 91.595 | -5.043 | 2.31E-06 | *** |

Signif. codes: 0 '***' 0.001 '**' 0.01 '*' 0.05 '.' 0.1 ' ' 1

Type II Analysis of Variance Table with Satterthwaite's method:

| Factor | Sum Sq | Mean Sq | NumDF | DenDF | F value | p value | Significance |
| --- | --- | --- | --- | --- | --- | --- | --- |
| treatment | 5598 | 2798.8 | 2 | 92.613 | 4.4395 | 0.01442 | * |
| time | 33565 | 11188.4 | 3 | 91.518 | 17.7473 | >0.00001 | *** |

Signif. codes: 0 '***' 0.001 '**' 0.01 '*' 0.05 '.' 0.1 ' ' 1

Pairwise comparison of time

| Contrast | Estimate | SE | df | t ratio | p value |
| --- | --- | --- | --- | --- | --- |
| baseline - post etorphine | 24.5 | 8.37 | 91.0 | 2.924 | 0.0223 |
| baseline - post naltrexone | -10.7 | 8.64 | 91.4 | -1.234 | 0.6073 |
| baseline - post treatment | 34.8 | 6.91 | 91.4 | 5.039 | <0.0001 |
| post etorphine - post naltrexone | -35.1 | 8.64 | 91.4 | -4.066 | 0.0006 |
| post etorphine - post treatment | 10.4 | 6.91 | 91.4 | 1.499 | 0.4424 |
| post naltrexone - post treatment | 45.5 | 7.22 | 91.2 | 6.303 | <0.0001 |

Results are averaged over the levels of: treatment

Degrees-of-freedom method: kenward-roger

P value adjustment: Tukey method for comparing a family of 4 estimates

Pairwise comparison of treatment

| Contrast | Estimate | SE | df | t ratio | p value |
| --- | --- | --- | --- | --- | --- |
| Control - BIMU-8 | 11.68 | 6.24 | 93.2 | 1.873 | 0.1522 |
| Control - Vatinoxan | -6.39 | 6.01 | 91.4 | -1.063 | 0.5396 |
| BIMU-8 - Vatinoxan | -18.08 | 6.14 | 92.7 | -2.943 | 0.0113 |

Results are averaged over the levels of: time

Degrees-of-freedom method: kenward-roger

P value adjustment: tukey method for comparing a family of 3 estimates

**Body temperature**

**Model: lmer (BT~ treatment*time + (1|Sheep.ID), data=data)**

Random effects:

| Group | Effect | Variance | Std. Dev. |
| --- | --- | --- | --- |
| Sheep.ID | Intercept | 0.07418 | 0.2724 |
| Residual | — | 0.05045 | 0.2246 |

Fixed effects:

| Effects | Estimate | Std. Error | df | t value | Pr(>\|t\|) | Significance |
| --- | --- | --- | --- | --- | --- | --- |
| (Intercept) | 39.11667 | 0.14412 | 12.63899 | 271.41 | <.0001 | *** |
| treatmentBIMU-8 | -0.11667 | 0.12968 | 84.95392 | -0.9 | 0.3708 |  |
| treatmentVatinoxan | -0.15 | 0.12968 | 84.95392 | -1.157 | 0.2506 |  |
| timepost etorphine | -0.03333 | 0.12968 | 84.95392 | -0.257 | 0.7978 |  |
| timepost naltrexone | -0.11052 | 0.13638 | 84.99381 | -0.81 | 0.42 |  |
| timepost treatment | -0.22662 | 0.1067 | 84.95956 | -2.124 | 0.0366 | * |
| treatmentBIMU-8: timepost etorphine | 0.08333 | 0.18339 | 84.95392 | 0.454 | 0.6507 |  |
| treatmentVatinoxan:timepost etorphine | 0.08333 | 0.18339 | 84.95392 | 0.454 | 0.6507 |  |
| treatmentBIMU-8:timepost naltrexone | 0.04257 | 0.19304 | 85.01179 | 0.221 | 0.826 |  |
| treatmentVatinoxan:timepost naltrexone | -0.22282 | 0.18819 | 84.97488 | -1.184 | 0.2397 |  |
| treatmentBIMU-8:timepost treatment | 0.08535 | 0.1526 | 85.00355 | 0.559 | 0.5774 |  |
| treatmentVatinoxan:timepost treatment | -0.30671 | 0.15032 | 84.95676 | -2.04 | 0.0444 | * |

Signif. codes: 0 '***' 0.001 '**' 0.01 '*' 0.05 '.' 0.1 ' ' 1

Type III Analysis of Variance Table with Satterthwaite's method:

| Effect | Sum Sq | Mean Sq | NumDF | DenDF | F value | Pr(>F) | Significance |
| --- | --- | --- | --- | --- | --- | --- | --- |
| treatment | 1.04264 | 0.52132 | 2 | 85.02 | 10.334 | 9.61e-05 | *** |
| time | 2.02060 | 0.67353 | 3 | 84.97 | 13.351 | 3.22e-07 | *** |
| treatment:time | 0.67086 | 0.11181 | 6 | 84.98 | 2.216 | 0.0491 | * |

Pairwise comparison of time across treatment:

*Treatment = control*

| Contrast | Estimate | SE | df | t.ratio | p.value |
| --- | --- | --- | --- | --- | --- |
| baseline – post etorphine | 0.0333 | 0.130 | 85.0 | 0.257 | 0.9940 |
| baseline – post naltrexone | 0.1105 | 0.136 | 85.0 | 0.810 | 0.8494 |
| baseline – post treatment | 0.2266 | 0.107 | 85.0 | 2.124 | 0.1539 |
| post etorphine – post naltrexone | 0.0772 | 0.136 | 85.0 | 0.566 | 0.9419 |
| post etorphine – post treatment | 0.1933 | 0.107 | 85.0 | 1.812 | 0.2751 |
| post naltrexone – post treatment | 0.1161 | 0.114 | 85.0 | 1.014 | 0.7417 |

*Treatment = BIMU-8*

| Contrast | Estimate | SE | df | t.ratio | p.value |
| --- | --- | --- | --- | --- | --- |
| baseline – post etorphine | -0.0500 | 0.130 | 85.0 | -0.386 | 0.9804 |
| baseline – post naltrexone | 0.0679 | 0.136 | 85.0 | 0.498 | 0.9593 |
| baseline – post treatment | 0.1413 | 0.109 | 85.1 | 1.296 | 0.5683 |
| post etorphine – post naltrexone | 0.1179 | 0.136 | 85.0 | 0.864 | 0.8231 |
| post etorphine – post treatment | 0.1913 | 0.109 | 85.1 | 1.754 | 0.3027 |
| post naltrexone – post treatment | 0.0733 | 0.116 | 85.0 | 0.632 | 0.9214 |

*Treatment = Vatinoxan*

| Contrast | Estimate | SE | df | t.ratio | p.value |
| --- | --- | --- | --- | --- | --- |
| baseline – post etorphine | -0.0500 | 0.130 | 85.0 | -0.386 | 0.9804 |
| baseline – post naltrexone | 0.3333 | 0.130 | 85.0 | 2.570 | 0.0566 |
| baseline – post treatment | 0.5333 | 0.106 | 85.0 | 5.037 | <0.0001 |
| post etorphine – post naltrexone | 0.3833 | 0.130 | 85.0 | 2.956 | 0.0206 |
| post etorphine – post treatment | 0.5833 | 0.106 | 85.0 | 5.509 | <0.0001 |
| post naltrexone – post treatment | 0.2000 | 0.106 | 85.0 | 1.889 | 0.2405 |

Degrees-of-freedom method: kenward-roger

P value adjustment: tukey method for comparing a family of 4 estimates

Pairwise comparison of treatment across time:

*Baseline*

| Contrast | Estimate | SE | df | t.ratio | p.value |
| --- | --- | --- | --- | --- | --- |
| Control – BIMU-8 | 0.1167 | 0.1297 | 85.0 | 0.900 | 0.6420 |
| Control – Vatinoxan | 0.1500 | 0.1297 | 85.0 | 1.157 | 0.4822 |
| BIMU-8 – Vatinoxan | 0.0333 | 0.1297 | 85.0 | 0.257 | 0.9642 |

*Post etorphine*

| Contrast | Estimate | SE | df | t.ratio | p.value |
| --- | --- | --- | --- | --- | --- |
| Control – BIMU-8 | 0.0333 | 0.1297 | 85.0 | 0.257 | 0.9642 |
| Control – Vatinoxan | 0.0667 | 0.1297 | 85.0 | 0.514 | 0.8647 |
| BIMU-8 – Vatinoxan | 0.0333 | 0.1297 | 85.0 | 0.257 | 0.9642 |

*Post treatment*

| Contrast | Estimate | SE | df | t.ratio | p.value |
| --- | --- | --- | --- | --- | --- |
| Control – BIMU-8 | 0.0313 | 0.0805 | 85.2 | 0.389 | 0.9200 |
| Control – Vatinoxan | 0.4567 | 0.0760 | 85.0 | 6.008 | <0.0001 |
| BIMU-8 – Vatinoxan | 0.4254 | 0.0793 | 85.2 | 5.365 | <0.0001 |

*Post naltrexone*

| Contrast | Estimate | SE | df | t.ratio | p.value |
| --- | --- | --- | --- | --- | --- |
| Control – BIMU-8 | 0.0741 | 0.1430 | 85.1 | 0.518 | 0.8628 |
| Control – Vatinoxan | 0.3728 | 0.1364 | 85.0 | 2.733 | 0.0206 |
| BIMU-8 – Vatinoxan | 0.2987 | 0.1364 | 85.0 | 2.189 | 0.0788 |

Degrees-of-freedom method: kenward-roger

P value adjustment: tukey method for comparing a family of 4 estimates

**Supplementary material 4: Additional pulmonary arterial and systemic arterial blood pressure**

**
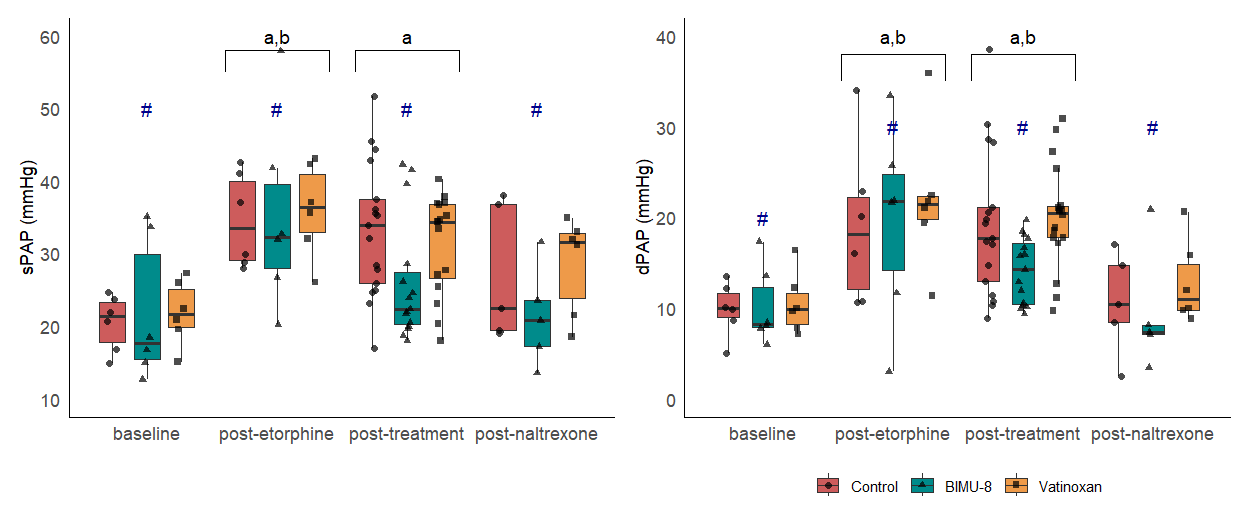
**

Figure 2: systolic and diastolic pulmonary arterial pressure were elevated after etorphine administration and remain high during immobilisation period. Similar to its effect on mPAP, BIMU-8 caused a reduction in sPAP and dPAP, while values in the Vatinoxan and Control groups remained elevated.

**
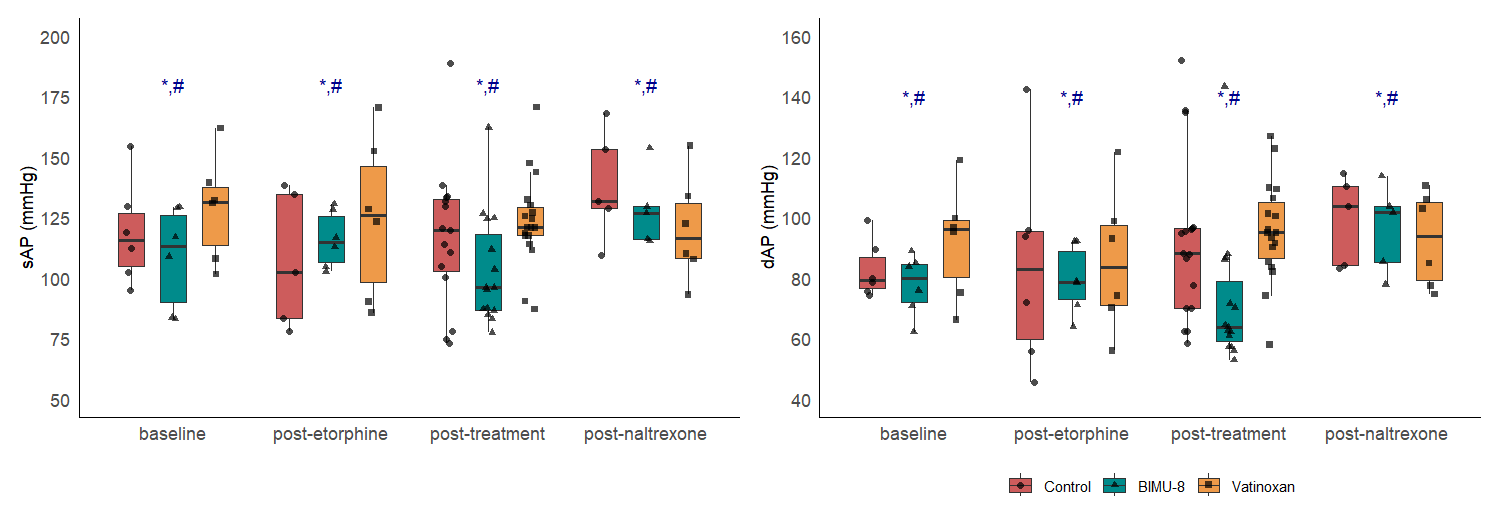
**

Figure 3: systolic and diastolic arterial blood pressure remained stable following etorphine administration. Similar to its effect on pulmonary arterial pressures, BIMU-8 led to a decrease in sAP and dAP without causing hypotension, while values in the Vatinoxan and Control groups remained unchanged from baseline.
